# *Terminalia chebula* decoction-processed *Aconitum kusnezoffii* preserves therapeutic effects through regulation of metabolism and intestinal microbiota

**DOI:** 10.3389/fmicb.2026.1879939

**Published:** 2026-08-04

**Authors:** Rina Sa, Li Mei, Gege Chen, Yasila Ba, Yongchang Bao, Liwei Guan, Rigen Te, Riliga Wu, Huricha Baigude, Lin Song

**Affiliations:** 1 College of Mongolian Medicine, Inner Mongolia Medical University, Hohhot, Inner Mongolia, China; 2 Medical Simulation Center, Inner Mongolia Medical University, Hohhot, Inner Mongolia, China; 3 Experimental Center, Inner Mongolia Traditional Chinese & Mongolian Medical Research Institute, Hohhot, Inner Mongolia, China; 4 School of Chemistry & Chemical Engineering, Inner Mongolia University, Hohhot, China; 5 Medical Innovation Center for Nationalities, Inner Mongolia Medical University, Hohhot, Inner Mongolia, China

**Keywords:** *A. kusnezoffii*, intestinal flora, metabolomics, pharmacodynamic effect, PI3K/AKT, *Terminalia chebula* decoction processing

## Abstract

**Background:**

*Aconitum kusnezoffii* Reichb. is a commonly used medicinal herb in Mongolian medicine (a branch of traditional Chinese medicine). Processing *A. kusnezoffii* with a decoction of *Terminalia chebula* Retz. represents a characteristic and widely applied detoxification method in Mongolian medicine practice. This processing technique can reduce the toxicity of *A. kusnezoffii*. However, the underlying mechanisms remain insufficiently understood.

**Methods:**

Normal Sprague–Dawley (SD) rats were randomly divided into the control (Ctrl), raw *A. kusnezoffii* (SCW), and *Terminalia chebula* decoction-processed *A. kusnezoffii* (HZZCW) groups. The metabolic profiles of rat feces and intestinal microbiota were detected. Hematoxylin and eosin (H&E) staining and enzyme-linked immunosorbent assay (ELISA) were performed to verify the toxicity of *A. kusnezoffii*. Western blot (WB) and RT-qPCR were used to assess the expression of the PI3K/AKT signaling pathway. In addition, a collagen-induced arthritis (CIA) model was established to evaluate the anti-inflammatory effect.

**Results:**

Metabolomic analysis revealed that processing *A. kusnezoffii* with *Terminalia chebula* decoction significantly altered metabolic pathways, particularly those related to ubiquinone biosynthesis, porphyrin and pyrimidine metabolism. The results of 16S rDNA sequencing showed that the processed product markedly changed the α-diversity of the gut microbiota and reduced the relative abundance of Xanthomonas and Corynebacterium. Pharmacodynamic evaluation indicated that HZZCW reduced the toxicity of raw *A. kusnezoffii* by regulating the levels of malondialdehyde (MDA), lactate dehydrogenase (LDH), and superoxide dismutase (SOD), while activating the PI3K/AKT pathway to maintain its therapeutic efficacy. Furthermore, the CIA model confirmed its significant anti-inflammatory activity.

**Conclusion:**

*Terminalia chebula* Decoction-processed *A. kusnezoffii* may exert its pharmacodynamic effects through multiple mechanisms, including modulation of ubiquinone biosynthesis, porphyrin metabolism, and pyrimidine metabolism, regulation of the intestinal microbiota, and activation of the PI3K/AKT signaling pathway.

## Graphical abstract

**Figure:**
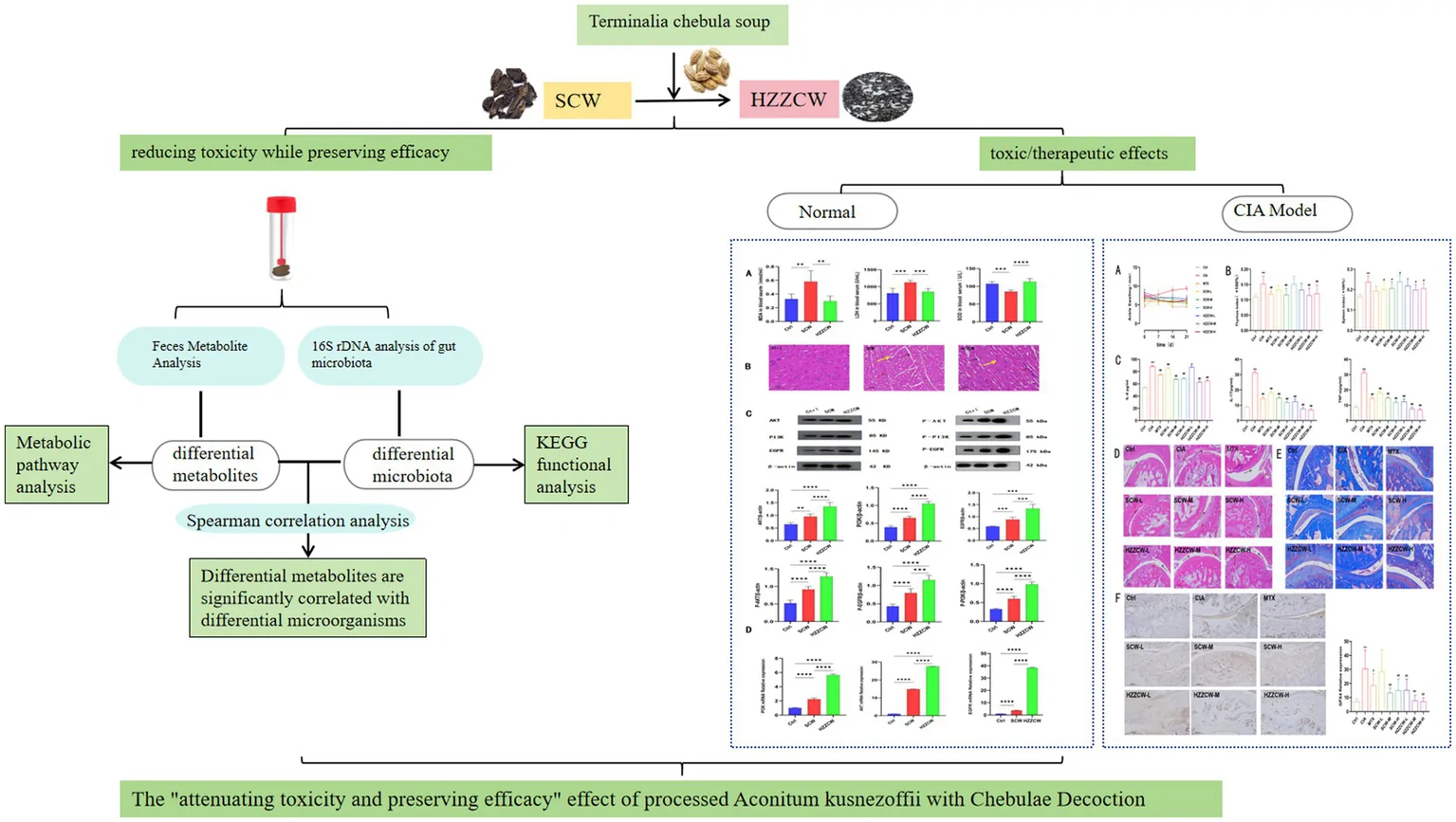

## Highlights

- Integrated analysis of fecal metabolomics and gut microbiota was conducted.
- Biomarkers related to *A. kusnezoffii* processed with *Terminalia chebula* decoction were identified.
- The combination of intestinal microbiota and metabolomics provides insights for traditional Chinese medicine processing.

## 1 Introduction

*Aconitum kusnezoffii* Reichb. is a representative Mongolian medicine characterized by its “dual properties of toxicity and efficacy.” It contains a variety of bioactive metabolites, including alkaloids, volatile oils, amino acids, and flavonoids. Clinically, it is used to reduce swelling, alleviate pain, eliminate “Xieriwusu” (a pathological condition associated with dampness accumulation in Mongolian medicine), and manage conditions such as rheumatoid arthritis, joint pain, and migratory stabbing pain. However, the presence of multiple toxic alkaloids limits its therapeutic application. Modern pharmacological studies have demonstrated that *A. kusnezoffii* exhibits anti-inflammatory, analgesic, anti-tumor, cardiotonic, antioxidant, and immunomodulatory effects, while also producing toxicological effects, including cardiotoxicity, neurotoxicity, reproductive toxicity, and embryotoxicity (Rong et al., 2021; Wu et al., 2021; Li, Yu S. et al., 2022). Consequently, research on *A. kusnezoffii* has focused on strategies to “reduce toxicity while preserving efficacy,” particularly through traditional processing methods. Processing *A. kusnezoffii* with *Terminalia chebula* decoction represents a distinctive and widely used approach in Mongolian medicine. Previous studies by the author’s team found that this method significantly reduces the levels of its primary toxic alkaloids—aconitine, mesaconitine, and hypaconitine (Zhang et al., 2016). *Terminalia chebula* Retz. or its variety *Terminalia chebula* Retz. var. *tomentella* Kurt. refers to the dried ripe fruit of the plant and contains bioactive metabolites, such as gallic acid, ellagic acid, corilagin, and chebulinic acid. Referred to as the “King of Mongolian Medicines,” it exhibits detoxifying properties, regulates the “Three Roots,” alleviates diarrhea, and removes pathogenic factors, in addition to anti-inflammation, anti-oxidation, and anti-tumor activities (Gupta et al., 2020; Prasad and Srivastava, 2020). Currently, research on *A. kusnezoffii* processed with *Terminalia chebula* decoction has predominantly focused on *in vitro* chemical constituent changes, while *in vivo* metabolic studies remain limited.

Variations in metabolite levels are a natural consequence of the onset and progression of many diseases, and these alterations often appear in biological fluids prior to the manifestation of clinical symptoms. Common biological samples used in metabolomic analyses include serum, plasma, urine, feces, and saliva, all of which contain abundant metabolite information (Yang, 2022). By analyzing the dynamic changes of metabolites in organisms, metabolomics can directly reflect pathophysiological states, providing a unique perspective for investigating disease mechanisms.

Metabolomics is characterized by integrity and systematicness when evaluating dynamic metabolic changes within organisms. This perspective is consistent with the holistic philosophy of Mongolian medicine and aligns with its therapeutic characteristics, including multi-target and multi-pathway regulation. The co-metabolic network between gut microbiota and the host plays an important role in the pathogenesis and treatment of various metabolic diseases (Wang Z. Y. et al., 2024). The gut microbiota contains abundant metabolic enzymes, and alterations in its composition can lead to corresponding changes in the metabolite profile. Moreover, metabolites secreted by intestinal microorganisms can modify the structure, metabolism, and pharmacological efficacy of drugs (Chen et al., 2023).

Therefore, to comprehensively identify the key biomarkers of *A. kusnezoffii* processed with *Terminalia chebula* decoction, this study employed untargeted metabolomics to analyze fecal metabolic profiles. Potential biomarkers and key metabolic pathways were screened. The findings were integrated with 16S rDNA sequencing data and pharmacodynamic evaluations to reveal the pharmacodynamic mechanisms of the processed *A. kusnezoffii* at the metabolomic and intestinal microbiota levels.

## 2 Materials and methods

### 2.1 Processing method

First, *Terminalia chebula* fruit (Huarong BenCao Chinese Medicinal Materials Co., Ltd., batch number 20220161), equivalent to half the weight of raw *A. kusnezoffii* Reichb (Inner Mongolia Kulun Mongolian Pharmaceutical Factory, batch number Y24072), was prepared. Ten times the volume of water was added, and the mixture was decocted for 1 h. The decoction was then filtered through four layers of gauze. Raw *A. kusnezoffii* was soaked in the filtrate for 48 h, followed by decoction for 8 h. Afterward, the processed *A. kusnezoffii* was removed, placed on a square plate, and dried at 60 °C.

The processing technology for Aconiti Radix Cocta processed with Chebulae Fructus decoction adopted in this experiment was an optimized and standardized procedure previously established in a National Natural Science Foundation of China project (Grant No. 30860391). Using the compatibility system of Mongolian medicines *Aconiti Radix* and Chebulae Fructus as the research object, the project employed the content of toxic diterpenoid alkaloids in *Aconiti Radix* and overall pharmacological activity as evaluation indicators. Key processing parameters, including the raw material ratio, soaking duration, decoction temperature, and decoction time, were analyzed using multi-index evaluation to determine the optimal processing conditions. The complete set of technologies has been filed as an invention patent (Application No. CN200910003515.6, Publication No. CN101564435), and related findings were awarded the Second Prize of Inner Mongolia Autonomous Region Natural Science Award in 2015.

Previous studies have elucidated the toxicity-reducing mechanism of this processing method. During co-soaking and decoction, highly toxic alkaloids in *Aconiti Radix*, such as aconitine, mesaconitine, and hypaconitine, undergo hydrolysis reactions, reducing inherent toxicity. Meanwhile, tannins abundant in Chebulae Fructus can form insoluble complexes with aconitum alkaloids, delaying intestinal absorption and further attenuating toxicity.

This study focuses on the *in vivo* differences in toxicity and efficacy between raw *Aconiti Radix* and *Aconiti Radix* processed with Chebulae Fructus decoction and the regulatory effects on the gut environment. This study does not involve the optimization of processing conditions. Therefore, the processed products were prepared using a fully validated optimal technology with fixed parameters. This approach minimizes variability arising from processing fluctuations and ensures the stability and comparability of experimental results between raw and processed medicinal materials.

### 2.2 Experimental animals

Male SPF Sprague–Dawley (SD) rats weighing 180–220 g were purchased from SPF (Beijing) Biotechnology Co., Ltd. (animal production license SCXK (Jing) 2021–0001). Animals were housed in an SPF-grade barrier facility under controlled conditions, including a temperature of 18–27 °C, relative humidity of 45–55%, and a12 h light/dark cycle with continuous mechanical ventilation. Standard rodent chow and sterile water were provided ad libitum. Bedding was replaced regularly, and all rats were acclimatized for 7 days prior to experimentation. All experimental procedures were approved by the Animal Ethics Committee of Inner Mongolia Medical University (Approval No. YKD202402144) and were conducted in strict accordance with the 3Rs principle and the ARRIVE 2.0 guidelines, as well as the national standard *Guidelines for Ethical Review of Laboratory Animal Welfare (GB/T 35892–2018)*.

### 2.3 Rationale for dose design

Dose gradients were established based on the LD₅₀ determined in this study. One-tenth, one-twentieth, and one-thirtieth of the LD₅₀ were designated as the high-, medium-, and low-dose levels, corresponding to administration doses of 0.237 g/kg, 0.118 g/kg, and 0.079 g/kg, respectively. This dose-graded design is widely applied in toxic-efficacy studies of traditional Chinese medicines. The high dose approaches the toxic threshold and is intended to fully characterize the inherent toxicity of raw Aconiti Radix. The medium and low doses span the range from mild toxicity to relatively safe exposure levels, allowing systematic evaluation of dose-dependent toxicological and pharmacological responses. The equal spacing of dose levels further facilitates quantitative analysis of dose-efficacy and dose-toxicity relationships.

The primary objective of this study was to investigate the effect of Chebulae Fructus decoction processing on the “toxicity reduction with retained efficacy” property of Aconiti Radix. To ensure experimental consistency and control for confounding effects of dose variation, identical low-, medium-, and high-dose regimens were applied to both raw and processed Aconiti Radix groups. A blank control group was also included to provide a physiological baseline, thereby establishing a comprehensive experimental design incorporating dose-gradient analysis and parallel comparisons between raw and processed materials.

### 2.4 Establishment of the CIA model and drug administration

**Figure 1:**
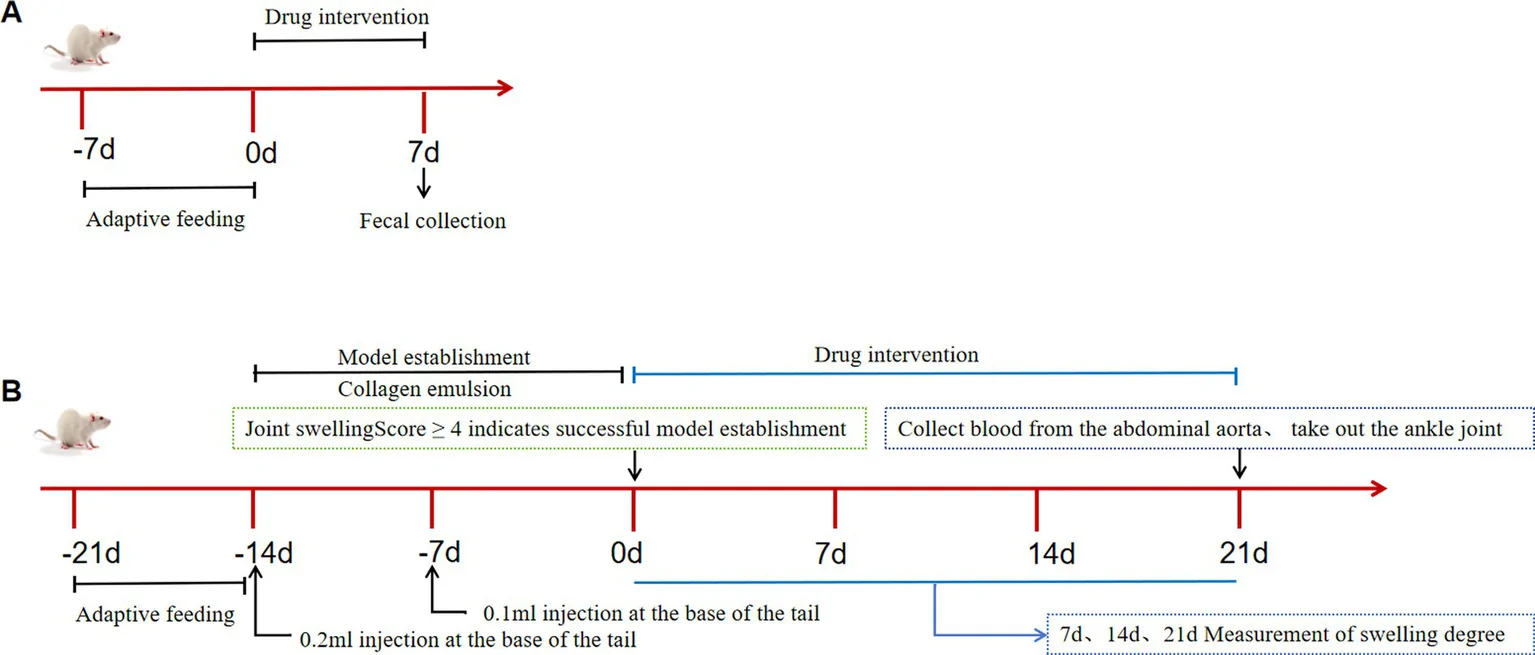
Timeline of all experimental procedures. **(A)** Experimental schedule for fecal sample collection; **(B)** Establishment protocol of collagen-induced arthritis(CIA) rat model and drug intervention timeline.

Eighteen male Sprague–Dawley (SD) rats were randomly divided into three groups: control (Ctrl), raw *A. kusnezoffii* (SCW), and *Terminalia chebula* decoction-processed *A. kusnezoffii* (HZZCW), with six rats in each group. The rats were administered the corresponding preparations at a dose of 0.237 g/kg for 7 days. According to preliminary acute toxicity tests, the LD50 was 2.3634 g/kg, and the experimental dose was set at 1/10 of the LD50. Fecal samples were collected from the rats within 0–24 h (Figure 1A).

Additionally, 63 male SD rats were used to establish the CIA model, with seven rats per group. The CIA model was induced using bovine type II collagen and incomplete Freund’s adjuvant. Briefly, 0.1 mL of collagen emulsion was injected into the root of the rat tail. Following booster immunization, the limbs of the rats were monitored for swelling. The model was considered successfully established when symptoms such as redness and swelling of limb joints appeared (Kang et al., 2025). The successfully modeled rats were randomly divided into eight groups: the model group (CIA), the methotrexate group (MTX, 1 mg/mL), the raw *A. kusnezoffii* low-, medium-, and high-dose groups (SCW-L, SCW-M, SCW-H, 0.079 g/kg, 0.118 g/kg, 0.237 g/kg, respectively), and the *Terminalia chebula* decoction-processed *A. kusnezoffii* low-, medium-, and high-dose groups (HZZCW-L, HZZCW-M, HZZCW-H, 0.079 g/kg, 0.118 g/kg, 0.237 g/kg, respectively). The normal control group and model group received 0.5% sodium CMC-Na solution. All treatments were administered intragastrically at a volume of 10 mL/kg. The MTX group was treated twice per week, whereas all other groups were treated once daily for three consecutive weeks (Figure 1B).

### 2.5 Detection of fecal metabolites

Approximately 25 mg of fecal samples were weighed into EP tubes under low-temperature conditions. Two homogenization beads and 500 μL of extraction solution (methanol:acetonitrile:water = 2:2:1, V/V) containing isotope-labeled internal standards were added. The samples were vortexed for 30 s, homogenized using a homogenizer (35 Hz, 4 min), and subsequently subjected to ultrasonic treatment in an ice-water bath for 5 min. The homogenization-sonication procedure was repeated three times. The samples were then incubated at −40 °C for 1 h.

Chromatographic conditions: Column: Chromatographic separation was performed using a Waters ACQUITY UPLC BEH Amide (2.1 mm × 50 mm, 1.7 μm). The mobile phases consisted of: Phase A: 25 mmol/L ammonium acetate and 25 mmol/L ammonium hydroxide dissolved in 1 L ultrapure water. Phase B: acetonitrile. The gradient elution program was as follows: 0–0.25 min: 95% B; 0.25–3.5 min: 95% → 65% B; 3.5–4 min: 65% → 40% B; 4–4.5 min: 40% B; 4.5–4.55 min: 40% → 95% B; 4.55–6 min: 95% B. The column temperature was maintained at 25 °C, the autosampler temperature at 4 °C, the flow rate at 0.5 mL/min, and the injection volume was 2 μL.

Mass spectrometric conditions: Mass spectrometric analysis was performed using an Orbitrap Exploris 120 mass spectrometer equipped with an electrospray ionization (ESI) source. Data were acquired in both positive and negative ionization modes. The main parameters were set as follows: Sheath gas flow: 50 arb; auxiliary gas flow: 15 arb; capillary temperature: 320 °C; MS resolution: 60,000; MS/MS resolution: 15,000.

### 2.6 Detection of intestinal microbiota

Genomic DNA was extracted using the TGuide S96 Magnetic Bead Soil/Fecal Genomic DNA Extraction Kit according to the manufacturer’s instructions. The composition of the intestinal flora was analyzed using 16S rDNA sequencing. The V3–V4 regions were amplified by polymerase chain reaction (PCR) with the forward primer 5′-ACTCCTACGGGAGGCAGCA-3′ and reverse primer 5’-GGACTACHVGGGTWTCTAAT-3′. Species annotation and taxonomic classification were conducted using databases such as Silva and NCBI. Alpha diversity analysis was performed using QIIME2 software.

### 2.7 Biochemical assays

Serum levels of malondialdehyde (MDA), superoxide dismutase (SOD), and lactate dehydrogenase (LDH) were measured using an automatic biochemical analyzer (Mindray Biomedical Electronics Co., Ltd., Shenzhen, China). The assay kits for MDA, SOD, and LDH were purchased from Shenzhen Youpin Biotechnology Co., Ltd., Shenzhen, China.

### 2.8 Hematoxylin and eosin (H&E) staining

Cardiac tissues were fixed in 4% paraformaldehyde, embedded in paraffin, and sectioned into 5 μm thick slices. After H&E staining, the sections were examined using an upright light microscope. For joint tissue analysis, rat ankle joints were fixed in 4% paraformaldehyde overnight and subsequently decalcified in 10% EDTA solution for 60 days. The tissues were then embedded in paraffin, sectioned into 5 μm slices, and subjected to H&E staining. Histopathological changes were observed under an upright light microscope.

### 2.9 Immunohistochemical (IHC) detection

Paraffin-embedded ankle joint sections were sequentially incubated with primary and secondary antibodies, followed by 3,3′-diaminobenzidine (DAB) chromogenic detection and hematoxylin staining. The stained sections were examined under an upright light microscope, and quantitative analysis was performed using ImageJ software. The glutathione peroxidase 4 (GPX4) antibody (catalog no. DF6701) used in this study was purchased from Affinity Biosciences.

### 2.10 Western blot analysis

Approximately 20 mg of tissue was homogenized in 250 μL of RIPA lysis buffer (Solarbio Science & Technology Co., Ltd., Beijing, China). The lysates were centrifuged at 12,000 × g for 15 min at 4 °C, and the supernatant was collected for further analysis. After concentration quantification, proteins were transferred onto polyvinylidene fluoride (PVDF) membranes (Merck KGaA, Darmstadt, Germany). The membranes were incubated with primary antibodies overnight at 4 °C, followed by incubation with secondary antibodies for 1 h at 37 °C. The antibodies used in this study included: EGFR (AF6043, 1:1000, Affinity Biosciences), PI3K (AF6242, 1:1000, Affinity Biosciences), AKT (AF6261, 1:1000, Affinity Biosciences), p-PI3K (AF3241, 1:1000, Affinity Biosciences), p-AKT (AF0016, 1:1000, Affinity Biosciences), p-EGFR (AF3048, 1:1000, Affinity Biosciences), and goat anti-rabbit IgG H&L secondary antibody (HRP-conjugated, SA00002-1, 1:10,000).

### 2.11 RT-qPCR

Total RNA was extracted from cardiac tissues using TransZol reagent (TransGen Biotech Co., Ltd., Beijing, China). Reverse transcription was performed using TransScript^®^ Uni All-in-One First-Strand cDNA Synthesis SuperMix for qPCR (with one-step gDNA removal) (TransGen Biotech Co., Ltd., Beijing, China) according to the manufacturer’s instructions. RT-qPCR was conducted using HiScript II Q RT SuperMix for qPCR (Vazyme Biotech Co., Ltd., Nanjing, China) on a CFX Connect™ system (Bio-Rad Laboratories (Shanghai) Co., Ltd.). The primer sequences used in this study are listed in Supplementary Table 1.

### 2.12 Masson staining

Paraffin-embedded ankle joint sections were dewaxed, followed by sequential staining with potassium dichromate, iron hematoxylin, and ponceau solution. The sections were then treated with phosphomolybdic acid and stained with aniline blue. Finally, the stained sections were observed under an upright light microscope.

### 2.13 Ankle joint measurement

The thickness of the right hind paw of each rat was measured using a digital vernier caliper with a precision of 0.01 mm to evaluate the degree of paw swelling.

### 2.14 Thymus index and spleen index

The thymus and spleen were carefully excised and weighed. The thymus index and spleen index were calculated according to the following formulas:

Thymus index = (thymus weight/body weight) × 100%.

Spleen index = (spleen weight/body weight) × 100%.

### 2.15 Enzyme-linked immunosorbent assay (ELISA)

ELISA kits for tumor necrosis factor-*α* (TNF-α), interleukin-6 (IL-6), and interleukin-17 (IL-17) came from Shanghai Enzyme-linked Biotechnology Co., Ltd., China. Standards and samples were prepared strictly according to the manufacturer’s instructions. Briefly, samples were added to the ELISA plates and incubated, followed by washing and incubation with the enzyme conjugate. After a second washing step, the substrate solution was added for color development, and the reaction was terminated with the stop solution. Absorbance was measured using a microplate reader, and the concentrations of target molecules were calculated.

### 2.16 Correlation analysis between metabolomics and gut microbiota

Spearman correlation analysis was performed to construct a microbiome-metabolite co-occurrence network. Strong correlation pairs were screened using the criteria |*r*| > 0.6 and *p* < 0.05, enabling the identification of potential interactions between key microbial taxa and differential metabolites.

### 2.17 Data analysis

Biological samples were analyzed using UPLC-Orbitrap Exploris 120-MS. Primary and secondary mass spectrometry data were acquired using Xcalibur software (version 4.4, Thermo Fisher Scientific). Raw data were converted to mzXML format using ProteoWizard software, and metabolite identification was performed using a custom R package in combination with the BiotreeDB database (V3.0). Multivariate statistical analyses, including principal component analysis (PCA) and orthogonal partial least squares discriminant analysis (OPLS-DA), were performed using SIMCA software (version 17.0) together with a self-developed R package. Metabolites were screened based on variable importance in projection (VIP) from the OPLS-DA model (VIP > 1), combined with T-test *p*-values and fold change (FC). Identified differential metabolites were further subjected to metabolic pathway enrichment analysis using MetaboAnalyst 6.0. Key metabolic pathways were screened using the criteria *p* < 0.05 and impact > 0.1. Statistical analyses were performed using SPSS 22.0, and figures were generated using GraphPad Prism 10.0. Data conforming to a normal distribution were expressed as mean ± standard deviation. Differences among groups were analyzed using one-way analysis of variance (ANOVA). When variances were homogeneous, the LSD test was used for pairwise comparisons; when variances were heterogeneous, rank-sum tests were applied. Comparisons between two groups were performed using the Wilcoxon rank-sum test, while comparisons among multiple groups were conducted using the Kruskal–Wallis test. A *p* < 0.05 was considered statistically significant.

## 3 Results

### 3.1 Data quality evaluation

The total ion chromatograms of fecal metabolites and quality control (QC) samples from the three groups of rats under both positive and negative ion modes are shown in Supplementary Figure 1. All samples exhibited strong signal intensity, with highly consistent peak areas and retention times. These results indicate good instrument stability, confirming that the metabolomic data are reliable and suitable for subsequent analysis.

### 3.2 Analysis of fecal metabolites

#### 3.2.1 Perform PCA and OPLS-DA analysis

**Figure 2:**
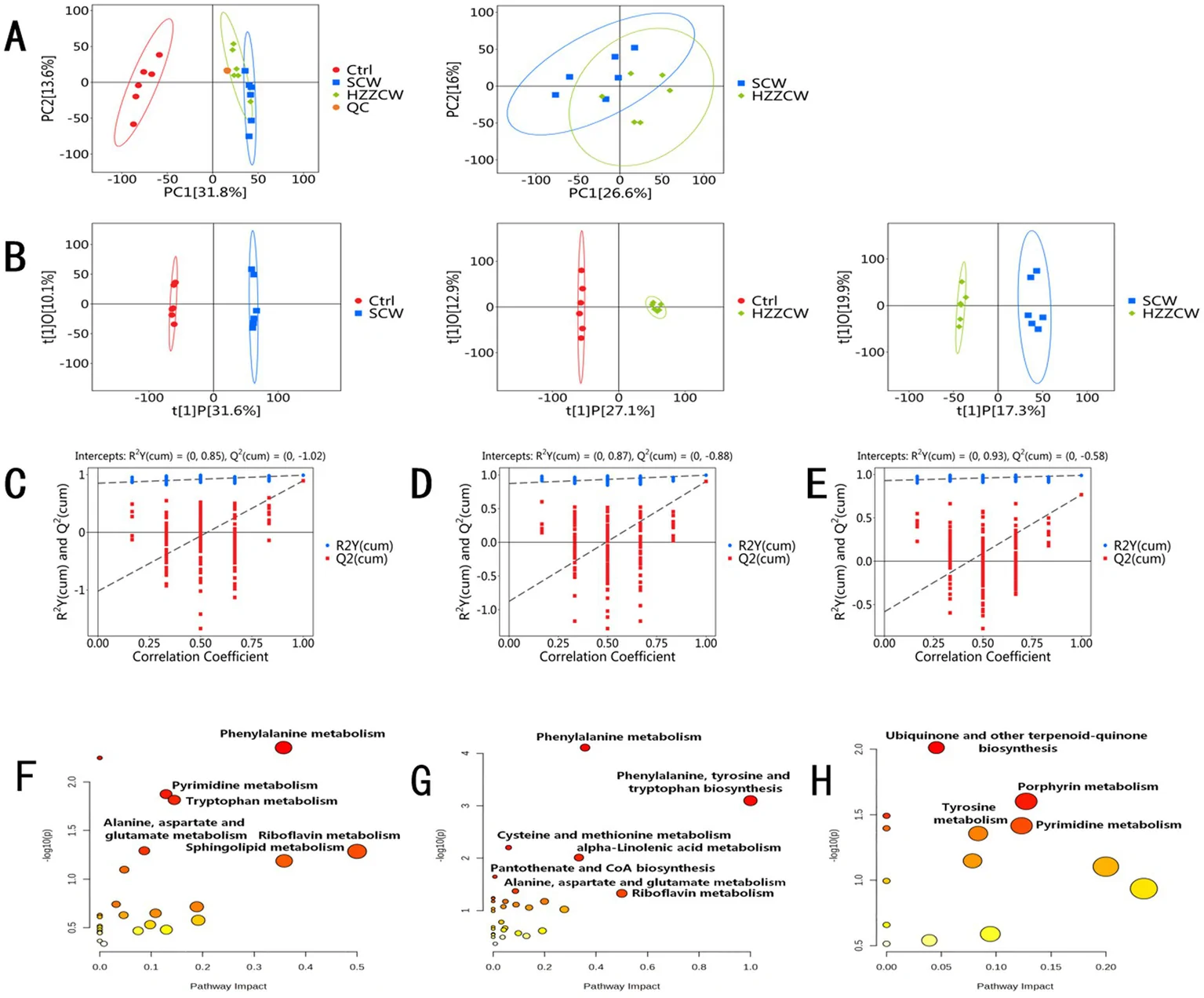
Fecal metabolic profiles and metabolic pathway analysis of the three groups. **(A)** PCA plot of fecal metabolites from the three groups and quality control (QC) samples, showing clear clustering between each group with small intra-group differences. **(B)** OPLS-DA plot of fecal metabolites from the three groups, indicating clear separation between the SCW and HZZCW groups. **(C)** Permutation test of the OPLS-DA model for the comparison between the control group and the SCW group. **(D)** Permutation test of the OPLS-DA model for the comparison between the control group and the HZZCW group. **(E)** Permutation test of the OPLS-DA model for the comparison between the SCW group and the HZZCW group. **(F)** Metabolic pathway enrichment analysis of differential metabolites in the SCW group. **(G)** Metabolic pathway enrichment analysis of differential metabolites in the HZZCW group. **(H)** Fecal metabolic pathways of differential metabolites between the two groups.

The fecal metabolites of the three groups were clearly clustered with minimal overlap, indicating significant differences in fecal metabolic profiles between the treatment groups and the control group. Comparison between the SCW and HZZCW groups showed distinct clustering without obvious separation, suggesting that both treatments altered the metabolic profile (Figure 2A). In the OPLS-DA score plots, the three groups displayed clear separation with small intra-group variation (Figure 2B). For Ctrl vs. SCW, the R^2^ and Q^2^ values were 0.85 and −1.02, respectively (Figure 2C); for Ctrl vs. HZZCW, the *R*^2^ and *Q*^2^ values were 0.87 and −0.88, respectively (Figure 2D); and for SCW vs. HZZCW, the R^2^ and Q^2^ values were 0.93 and −0.58, respectively (Figure 2E). These results indicate that the model is robust and free from overfitting and possesses good predictability, demonstrating statistically significant differences among groups.

#### 3.2.2 Differential metabolite analysis

**Figure 3:**
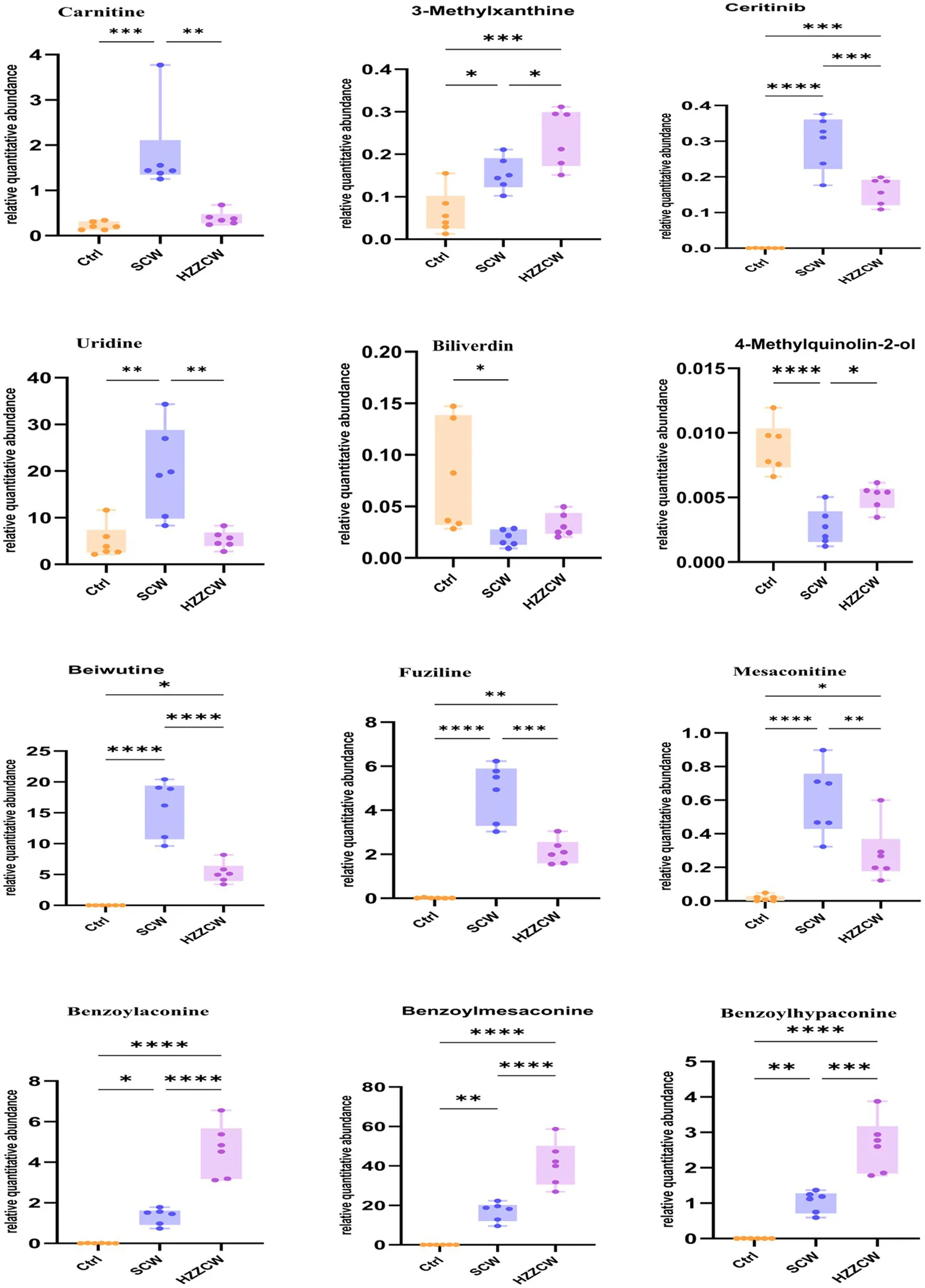
Changes in the contents of 12 differential metabolites. Data are expressed as mean ± standard error and analyzed by one-way ANOVA.*p < 0.05, **p < 0.01, ***p < 0.001, ****p < 0.0001.

The volcano plots of differential metabolites are shown in Supplementary Figures 2A–C. A total of 231 metabolites were upregulated, and 232 metabolites were downregulated in the SCW group; 226 metabolites were upregulated, and 191 metabolites were downregulated in the HZZCW group. Compared with the SCW group, 206 metabolites were upregulated, and 122 metabolites were downregulated in the HZZCW group. Based on the screening criteria, a total of 62 differential metabolites were identified in the SCW group; 34 upregulated and 28 downregulated (Supplementary Table 2-1). In the HZZCW group, 78 differential metabolites were identified: 51 upregulated and 27 downregulated (Supplementary Table 2-2). In HZZCW vs. SCW, 27 upregulated and 16 downregulated (Supplementary Table 2-3). A total of 12 overlapping metabolites were identified among the three groups, forming a subset of variables associated with *Terminalia chebula* decoction-processed *A. kusnezoffii* (Table 1). Compared with the Ctrl group, the SCW group showed significantly elevated relative levels of 10 metabolites, including fuziline, while the levels of two metabolites (biliverdin and 4-methylquinolin-2-ol) were markedly decreased (*p* < 0.05). In the HZZCW group, the relative levels of eight metabolites, including fuziline, mesaconitine, beiwutine, Benzoylaconine, and Benzoylhypaconine, were significantly increased compared with the Ctrl group (*p* < 0.05). Compared with the SCW group, the HZZCW group showed notably reduced relative levels of six metabolites, including fuziline, mesaconitine, and beiwutine (*p* < 0.01), while benzoylaconine, benzoylmesaconine, and benzoylhypaconine were significantly higher in the HZZCW group (Figure 3). These results suggest that the 12 overlapping metabolites may serve as potential fecal metabolic biomarkers associated with HZZCW treatment.

**Table 1:** Information on 13 significantly changed metabolites among the Ctrl, SCW, and HZZCW groups.

No.	Model	Specific name	m/z	rt (min)	Ctrl vs. SCW			Ctrl vs. HZZCW			SCW vs. HZZCW		
					VIP	FC	*P*-value	VIP	FC	*P*-value	VIP	FC	*P*-value
1	ESI+	Carnitine	162.1115	3.3833	1.8873	8.3101	0.0099	–	–	–	2.3929	0.2151	0.0152
2	ESI+	Fuziline	454.2773	1.1417	3.2392	313.7724	0.0003	3.2860	137.8313	0.0002	1.6691	0.4393	0.0009
3	ESI+	Mesaconitine	632.3032	0.4183	2.6812	35.5666	0.0011	2.5944	16.7119	0.0122	1.5169	0.4699	0.0171
4	ESI-	Uridine	243.0614	1.1283	1.4386	4.1018	0.0119	–	–	–	1.9831	0.2680	0.0148
5	ESI-	Biliverdin	581.2384	2.4200	1.2725	0.2497	0.0450	–	–	–	1.0859	1.6521	0.0494
6	ESI+	Benzoylhypaconine	574.2978	1.0350	3.3920	428.2428	0.0004	3.9740	1081.9900	0.0004	1.7605	2.5266	0.0008
7	ESI+	Beiwutine	648.2974	0.4983	4.6111	6521.2128	0.0004	4.7884	2168.4803	0.0006	1.9869	0.3325	0.0014
8	ESI+	Benzoylaconine	604.3079	1.1733	3.0364	161.8056	0.0004	3.7084	556.5139	0.0004	2.0968	3.4394	0.0012
9	ESI+	Benzoylmesaconine	590.2918	1.2583	3.5377	1063.2761	0.0003	4.1161	2593.3079	0.0003	1.7251	2.4390	0.0007
10	ESI-	3-Methylxanthine	165.0413	1.8567	1.1911	2.4492	0.0062	1.6700	3.8348	0.0005	1.1379	1.5657	0.0221
11	ESI+	Ceritinib	558.2303	0.4700	3.9231	1067.4680	0.0002	4.1249	578.0174	0.0001	1.3471	0.5415	0.0028
12	ESI+	4-Methylquinolin-2-ol	160.0747	0.8700	1.4216	0.3029	0.0001	–	–	–	1.4789	1.8770	0.0068

#### 3.2.3 Differential metabolic pathway analysis

Metabolic pathway analysis showed that the differential metabolites of the SCW group were involved in a total of 6 metabolic pathways, including phenylalanine metabolism, pyrimidine metabolism, tryptophan metabolism, alanine, aspartate, and glutamate metabolism, riboflavin metabolism, and sphingolipid metabolism (Figure 2F). The differential metabolites of the HZZCW group were involved in a total of 7 metabolic pathways, including phenylalanine metabolism, phenylalanine, tyrosine, and tryptophan biosynthesis, alpha-linolenic acid metabolism, cysteine and methionine metabolism, pantothenate and CoA biosynthesis, alanine, aspartate, and glutamate metabolism, and riboflavin metabolism (Figure 2G). In SCW vs. HZZCW, the differential metabolites were associated with 4 metabolic pathways, namely ubiquinone and other terpenoid-quinone biosynthesis, porphyrin metabolism, tyrosine metabolism, and pyrimidine metabolism (Figure 2H).

### 3.3 Analysis of intestinal microbial structure

#### 3.3.1 Alpha diversity analysis

**Figure 4:**
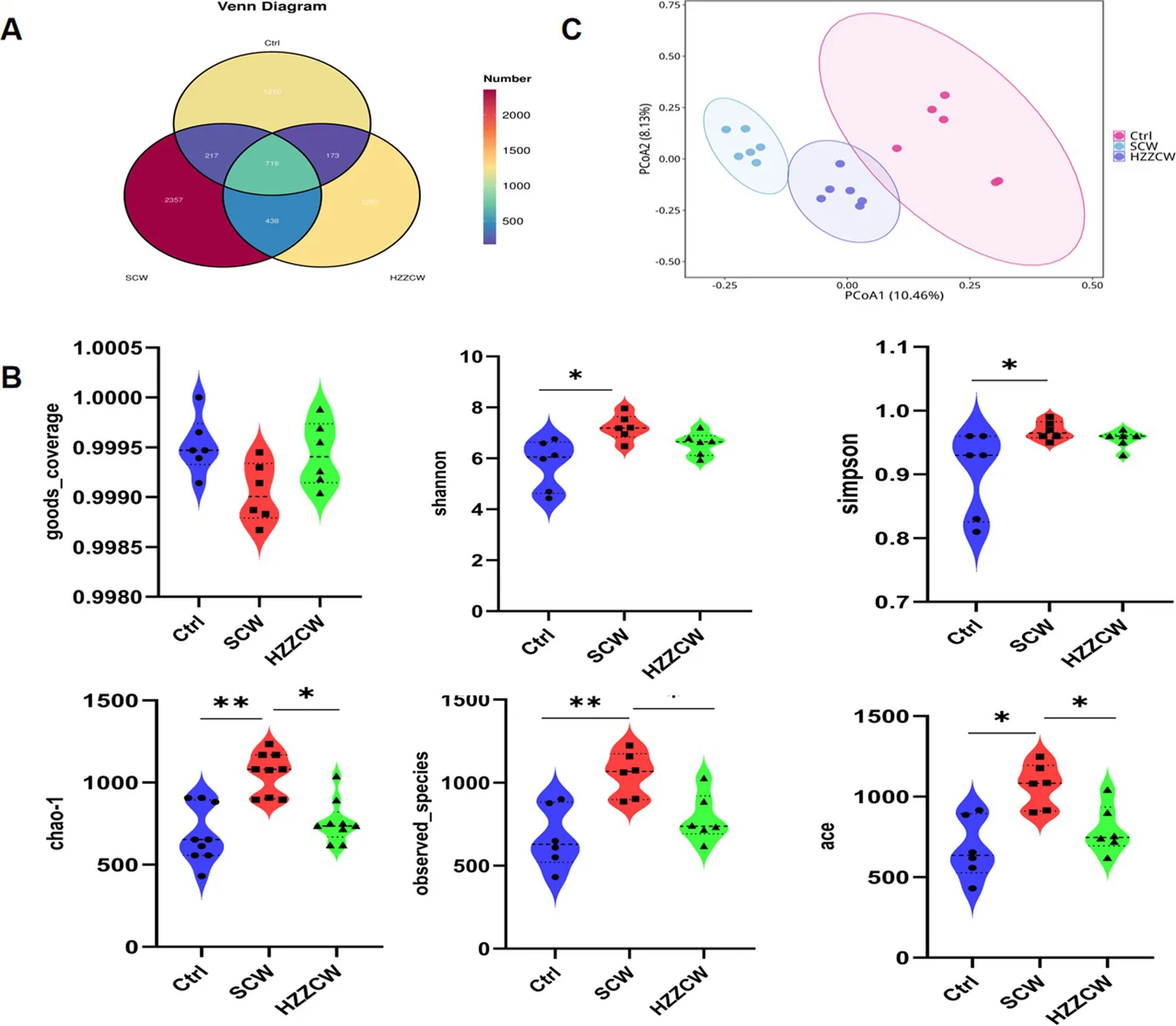
Effects of SCW and HZZCW on intestinal microbial diversity and microbial composition. **(A)** Shared and unique OTUs among the three groups. **(B)** Alpha diversity of intestinal microbiota, and PCA of intestinal microorganisms **(C)**. Data are expressed as mean ± standard error and analyzed by one-way ANOVA. *p < 0.05, **p < 0.01.

The Venn diagram showed that a total of 4,824 operational taxonomic units (OTUs) were identified among the three groups, among which 716 OTUs (14.84%) were shared by all groups. The SCW group shared 19.34% of its OTUs with the Ctrl group, while the HZZCW group shared 23.92% of its OTUs with the Ctrl group, indicating that both SCW and HZZCW altered the composition of intestinal microbial species, although the effect of HZZCW was relatively smaller (Figure 4A). Alpha diversity analysis was performed to evaluate the abundance and diversity of microbial communities. The Observed species index, Chao1 index, and ACE index estimate the total number of OTUs in the microbial community using two algorithms, primarily reflecting species richness within samples (Li et al., 2025). In contrast, the Shannon index and Simpson index capture the richness and evenness of species in the community (Yuan et al., 2021). The Good’s coverage index represents the sequencing depth of the samples. In this study, the sequencing coverage exceeded 0.99 for all samples, indicating that the probability of undetected sequences was low and that the sequencing depth was sufficient for reliable analysis. Compared with the Ctrl group, the observed species, Shannon, Chao1, and Ace indices in the SCW group were significantly increased (*p* < 0.01; *p* < 0.05), while the Simpson and Good’s coverage indices were substantially decreased (*p* < 0.05). However, no significant changes were observed after HZZCW administration (Figure 4B).

#### 3.3.2 Beta diversity analysis

**Figure 5:**
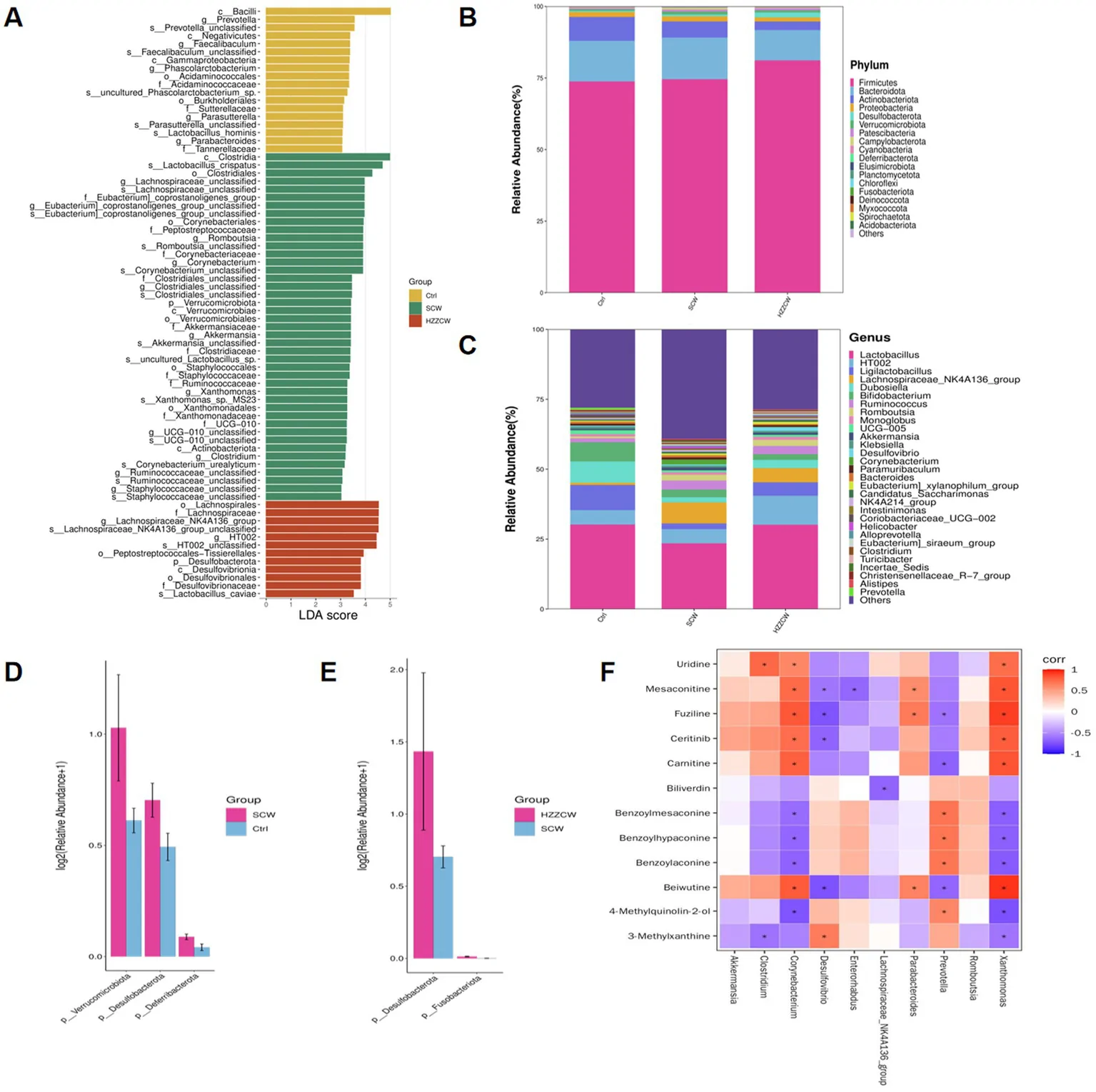
Effects of SCW and HZZCW on intestinal microbial diversity and microbial composition. **(A)** LEfSe analysis. **(B)** Relative abundance of intestinal microbiota at the phylum level. **(C)** Relative abundance of intestinal microbiota at the genus level. **(D)** Differential bacterial phyla between SCW and Ctrl at the phylum level (*p* < 0.05). **(E)** Differential bacterial phyla between SCW and HZZCW at the phylum level (*p* < 0.05). **(F)** Spearman correlation analysis showing the relationships among *Terminalia chebula* decoction-processed *A. kusnezoffii*, fecal metabolites, and intestinal microorganisms. Correlation coefficients are represented by color gradients, where red indicates negative correlation and blue indicates positive correlation. *p < 0.05.

Beta diversity analysis revealed differences in microbial community structure between the treatment and Ctrl groups. The SCW group was clearly separated from the Ctrl group, whereas the HZZCW group clustered closer to the Ctrl group, suggesting that raw *A. kusnezoffii* significantly altered intestinal microbial diversity, whereas the effect of *Terminalia chebula* decoction-processed *A. kusnezoffii* was comparatively weaker (Figure 4C). Linear discriminant analysis (LDA) further identified key microbial taxa responsible for group differences. At the phylum level, the SCW group was characterized by *Corynebacterium urealyticum* and *Xanthomonas MS23*, while *Lactobacillus caviae* was identified as a characteristic species in the HZZCW group. At the genus level, the SCW group was mainly characterized by *Akkermansia* and *Xanthomonas*, while HT002 and *Lachnospiraceae_NK4A136_group* were the dominant genera in the HZZCW group (Figure 5A). A stacked bar chart illustrating the top 30 most abundant taxa revealed differences in microbial composition among the three groups. At the phylum level, the levels of *Desulfobacterota*, *Verrucomicrobiota*, and *Deferribacterota* were significantly increased in the SCW group (Figures 5B,D). In the HZZCW group, *Desulfobacterota* and *Verrucomicrobiota* were significantly increased, but the increase in *Verrucomicrobiota* was less pronounced. Compared with the SCW group, the HZZCW group showed significantly higher levels of *Desulfobacterota* and *Fusobacteriota* (Figures 5B,E and Supplementary Figure 3B). At the genus level, compared with the Ctrl group, the relative abundances of *Lachnospiraceae_NK4A136_group*, *Akkermansia*, *Corynebacterium*, and *Xanthomonas* were significantly increased in the SCW group (*p* < 0.05), whereas the relative abundance of *Prevotella* was significantly reduced (*p* < 0.05). In the HZZCW group, the relative abundances of *Xanthomonas* and *Corynebacterium* were significantly reduced (*p* < 0.05), and the relative abundance of *Xanthomonas* was restored to the normal level (Figure 5C and Supplementary Figure 3A). These results suggest that processing *A. kusnezoffii* with *Terminalia chebula* decoction modifies its impact on intestinal microbial composition.

#### 3.3.3 Correlation analysis and functional analysis

To explore the potential associations between gut microbiota and differential metabolites, Spearman correlation analysis was performed between the top 10 intestinal microbial genera and 12 fecal differential metabolites, as shown in Figure 5F. The screening criteria for microbiota and metabolites were as follows. First, low-abundance taxa are more susceptible to sequencing noise and may generate spurious correlations. Therefore, they were excluded to improve the reliability of correlation analyses. Second, the top 10 genera ranked by average relative abundance were selected as representative of the core intestinal microbiota. These taxa are involved in key host metabolic processes, including short-chain fatty acid, bile acid, polysaccharide, and amino acid metabolism. Therefore, they are particularly informative for elucidating microbe-metabolite interaction patterns. Third, restricting the number of taxa enhances the clarity of visualization and facilitates the intuitive identification of key microbe-metabolite correlations. *Clostridium* showed a positive correlation with Uridine (Cor = 0.75) and a negative correlation with 3-Methylxanthine (Cor = −0.71). *Corynebacterium* was positively correlated with Uridine (Cor = 0.71), Mesaconitine (Cor = 0.74), Fuziline (Cor = 0.81), Ceritinib (Cor = 0.73), Carnitine (Cor = 0.78), and Beiwutine (Cor = 0.80) and negatively correlated with Benzoylhypaconine (Cor = −0.66), Benzoylaconine (Cor = −0.68), Benzoylmesaconine (Cor = −0.64), and 4-Methylquinolin-2-ol (Cor = −0.74). *Desulfovibrio* showed negative correlations with Mesaconitine (Cor = −0.59), Fuziline (Cor = −0.74), Ceritinib (Cor = −0.67), and Beiwutine (Cor = −0.75), while it was positively correlated with 3-Methylxanthine (Cor = 0.66). *Enterorhabdus* was negatively correlated with Mesaconitine (Cor = −0.66). *Lachnospiraceae_NK4A136_group* exhibited a negative correlation with Biliverdin (Cor = −0.67). *Parabacteroides* was positively correlated with Mesaconitine (Cor = 0.59), Fuziline (Cor = 0.67), and Beiwutine (Cor = 0.63). *Prevotella* showed negative correlations with Fuziline (Cor = −0.61), Carnitine (Cor = −0.68), and Beiwutine (Cor = −0.66) and positive correlations with Benzoylhypaconine (Cor = 0.69), Benzoylaconine (Cor = 0.69), Benzoylmesaconine (Cor = 0.70), and 4-Methylquinolin-2-ol (Cor = 0.61). *Xanthomonas* was positively correlated with Uridine (Cor = 0.73), Mesaconitine (Cor = 0.82), Fuziline (Cor = 0.90), Ceritinib (Cor = 0.79), Carnitine (Cor = 0.82), and Beiwutine (Cor = 0.93), while negative correlations were observed with Benzoylhypaconine (Cor = −0.69), Benzoylaconine (Cor = −0.71), Benzoylmesaconine (Cor = −0.70), 4-Methylquinolin-2-ol (Cor = −0.75), and 3-Methylxanthine (Cor = −0.60). These findings suggest that the detoxification effect of *A. kusnezoffii* processed with *Terminalia chebula* decoction may be closely associated with alterations in intestinal microorganisms and their metabolism.

**Figure 6:**
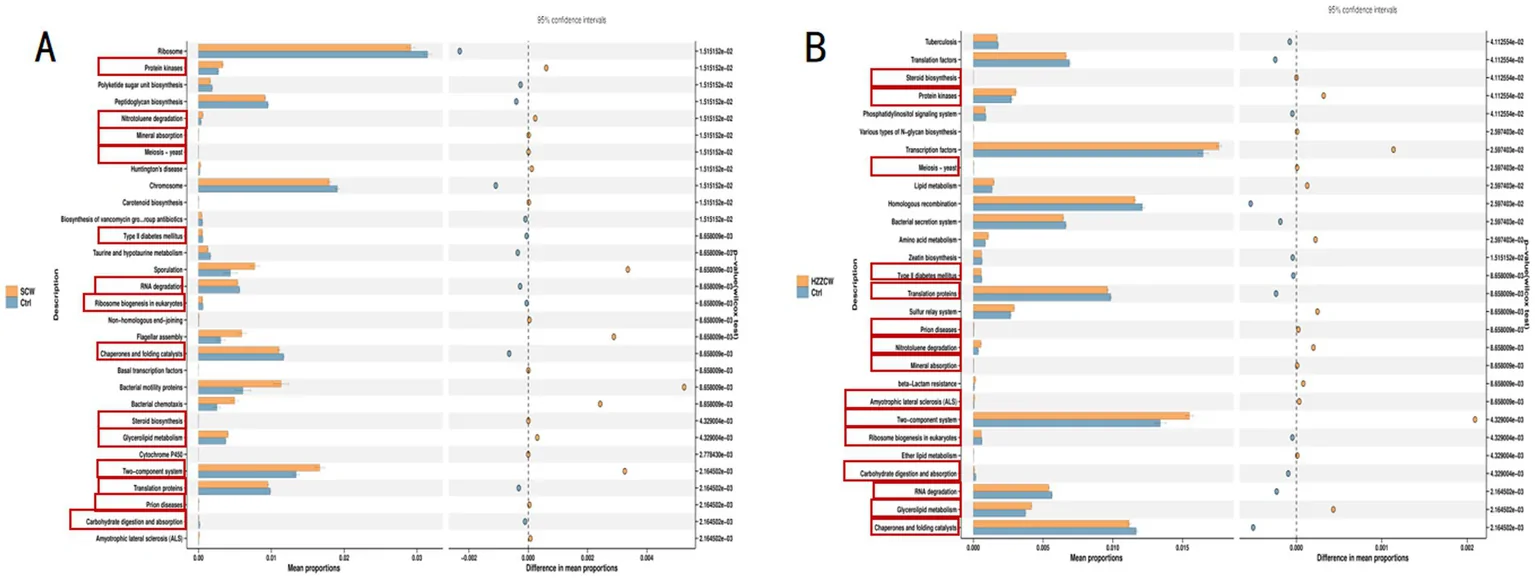
KEGG functional analysis of differential flora.

To further explore the functional characteristics of the microbial communities, KEGG analysis was conducted. Compared with the Ctrl group, the SCW and HZZCW groups displayed significant upregulation of metabolic functions, including Protein kinases, Mineral absorption, Steroid biosynthesis, Meiosis-yeast, Two-component system, Prion diseases, and Amyotrophic lateral sclerosis (ALS) (*p* < 0.01). In contrast, the functions of Type II diabetes mellitus, RNA degradation, Ribosome biogenesis in eukaryotes, Chaperones and folding catalysts, Translation proteins, and Carbohydrate digestion and absorption were significantly downregulated (*p* < 0.01) (Figures 6A,B).

#### 3.3.4 Correlation analysis between gut microbiota and functional pathways

**Figure 7:**
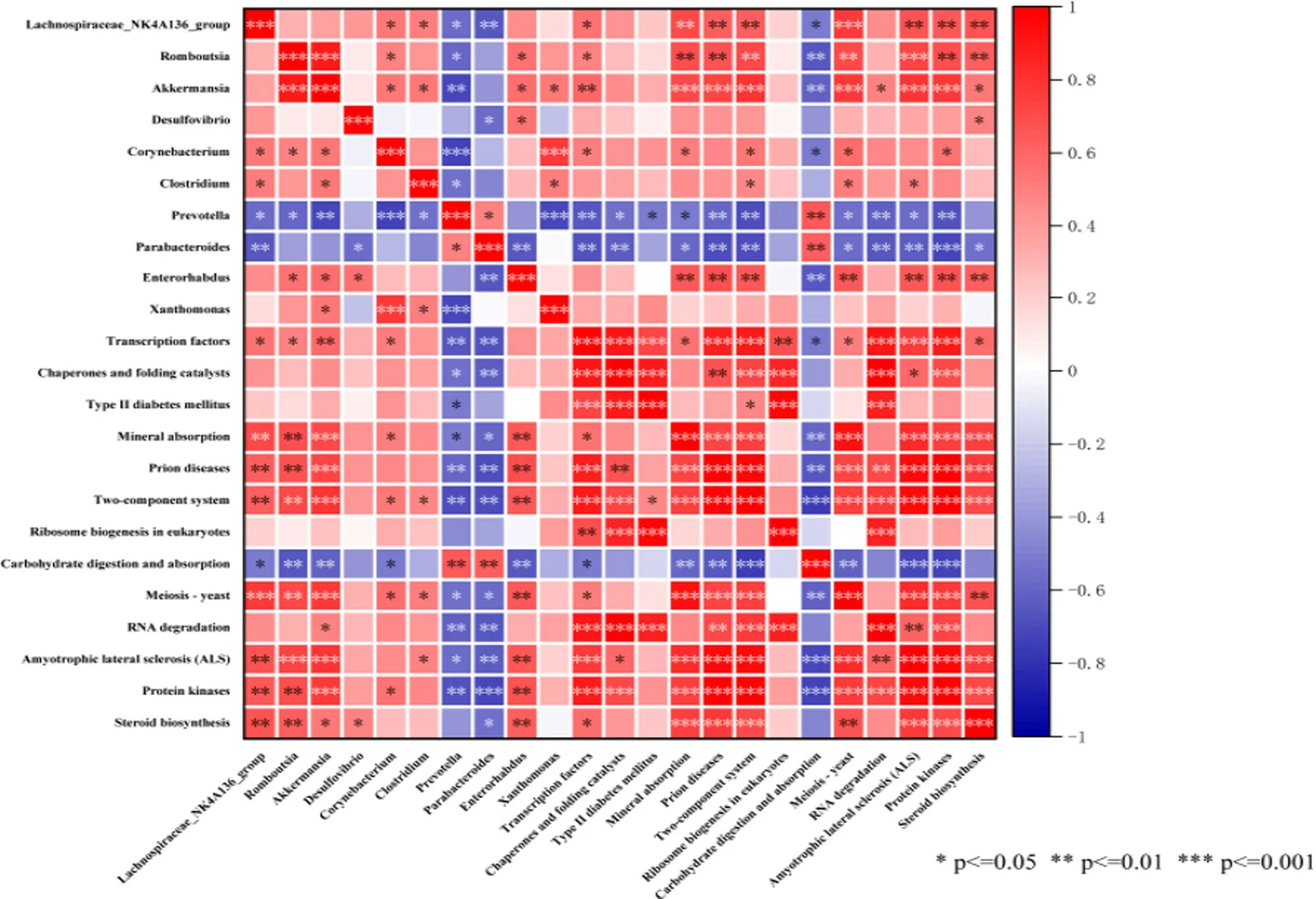
Spearman correlation analysis between KEGG pathways and differential genera.

Correlation analysis showed that the relative abundance of *Corynebacterium* was significantly and positively correlated with the enrichment of the protein kinase pathway (*r* = 0.55, *p* < 0.05) and Mineral absorption (*r* = 0.52, *p* < 0.05). Intervention with raw *Aconitum kusnezoffii* increased the abundance of *Corynebacterium*, accompanied by enhanced overall protein kinase function in the gut microbiota. By contrast, processing with *Chebulae Fructus* decoction reduced the abundance of this genus and alleviated protein kinase pathway enrichment. No significant correlations were detected between *Xanthomonas* and either the protein kinase pathway or Mineral absorption (*p* > 0.05) (Figure 7).

### 3.4 Pharmacological effects of *Aconitum kusnezoffii* processed with *Terminalia chebula* decoction

**Figure 8:**
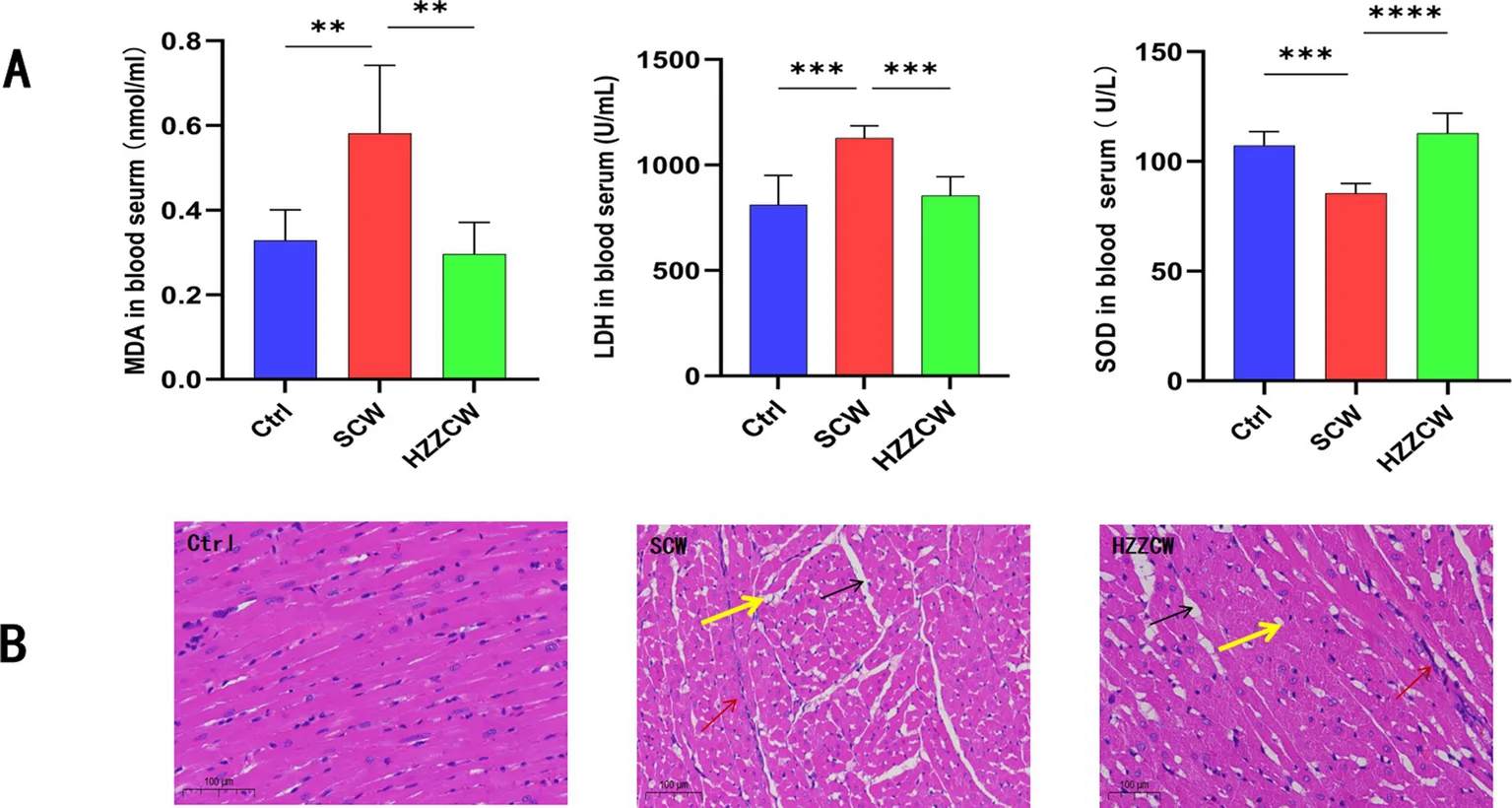
Effects of *Terminalia chebula* decoction-processed *A. kusnezoffii* on toxicity/efficacy. **(A)** Serum MDA, LDH, and SOD levels in rats (*n* = 6 per group). **(B)** Representative H&E staining images of cardiac tissue (scale bar: 100 μm). Data are expressed as mean ± standard error and analyzed by one-way ANOVA. **p < 0.01, ***p < 0.001, ****p < 0.0001.

**Figure 9:**
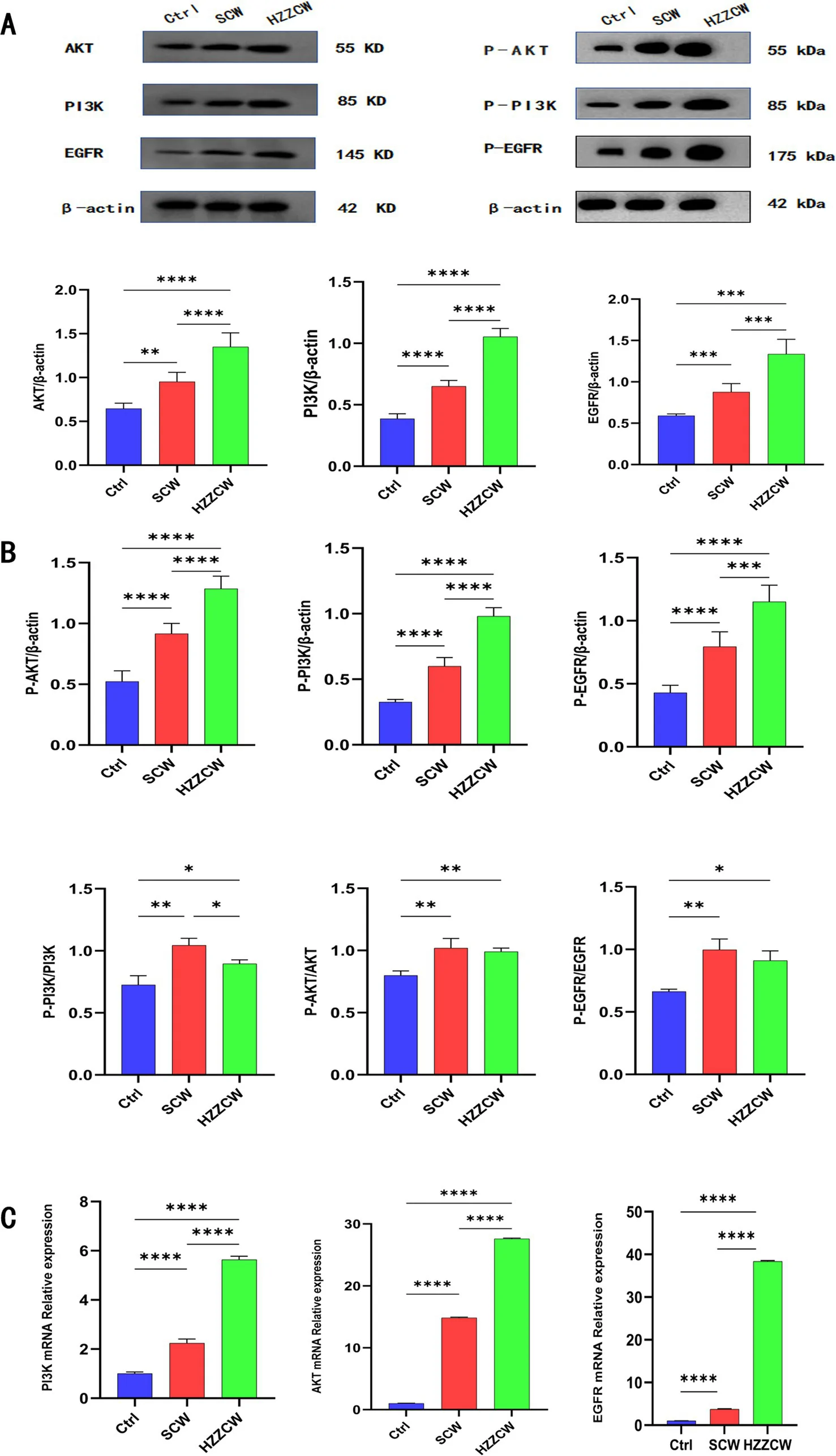
Effects of *Terminalia chebula* decoction-processed *A. kusnezoffii* on toxicity/efficacy. **(A)** Representative bands and quantitative analysis of AKT, PI3K, and EGFR protein expressions in cardiac tissue (*n* = 6 per group). **(B)** Representative bands and quantitative analysis of p-AKT, p-PI3K, and p-EGFR protein expressions in cardiac tissue (*n* = 6 per group). **(C)** Relative mRNA expression of AKT, PI3K, and EGFR in cardiac tissue (*n* = 6 per group). Data are expressed as mean ± standard error and analyzed by one-way ANOVA. *p < 0.05, **p < 0.01, ***p < 0.001, ****p < 0.0001.

Compared with the Ctrl group, the SCW group showed significantly increased serum MDA and LDH levels (*p* < 0.01) and significantly decreased SOD activity (*p* < 0.01) (Figure 8A). Pathological changes in cardiac tissue included disordered myocardial fibers, fragmentation, mild fibrous tissue hyperplasia, myocardial cell necrosis with deeply stained and pyknotic nuclei, and inflammatory cell infiltration in the myocardial interstitium (Figure 8B). In contrast, in the HZZCW group, no significant changes were observed in serum MDA, LDH, and SOD levels (*p* > 0.05). Histopathological analysis revealed disordered myocardial fibers, mild fibrous tissue hyperplasia, and a small number of necrotic myocardial cells with pyknotic nuclei, but no obvious inflammatory cell infiltration in the myocardial interstitium. These findings indicate that processing *A. kusnezoffii* with *Terminalia chebula* decoction effectively reduces its toxicity. Furthermore, compared with the Ctrl group, both protein and mRNA expression levels of AKT, PI3K, and EGFR were significantly upregulated in the SCW group (*p* < 0.01). These expression levels were further increased in the HZZCW group relative to the SCW group (*p* < 0.01) (Figures 9A,C). In addition, both treatment groups exhibited significantly higher phosphorylation ratios of p-AKT/AKT, p-PI3K/PI3K, and p-EGFR/EGFR compared with the Ctrl group (*p* < 0.01). Notably, the p-PI3K/PI3K ratio in the HZZCW group was significantly lower than that in the SCW group (*p* < 0.05) (Figure 9B). These findings indicate that Chebulae Fructus decoction-processed aconite retains its cardioprotective effect.

**Figure 10:**
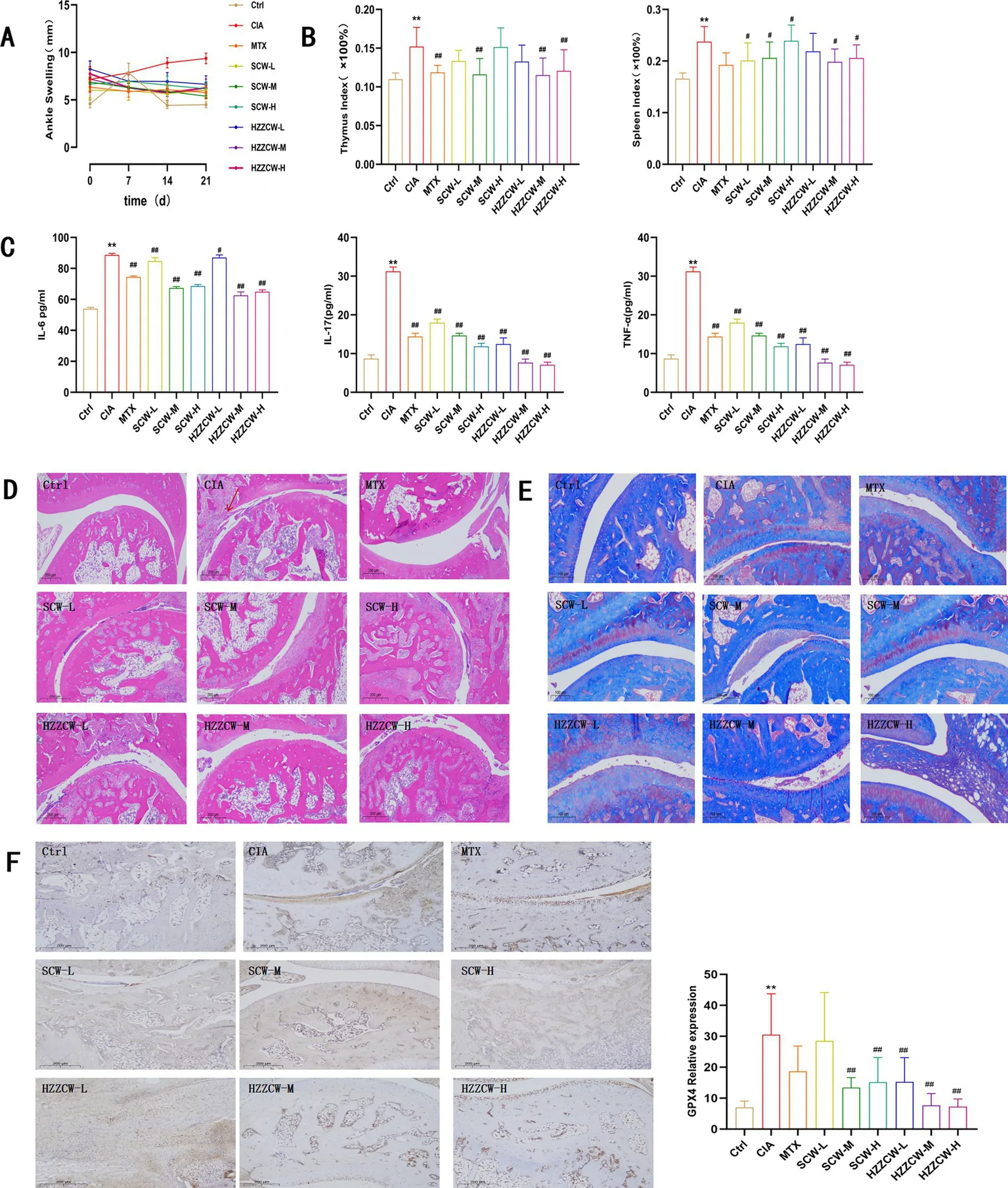
Effects of raw *A. kusnezoffii* and *Terminalia chebula* decoction-processed *A. kusnezoffii* on CIA model rats. **(A)** Ankle swelling measurements (*n* = 6). **(B)** Thymus and spleen indices (*n* = 6). **(C)** Serum IL-6, IL-17, and TNF-α levels (*n* = 7). **(D)** H&E staining of ankle joints (×40, scale bar: 100 μm). **(E)** Masson staining of ankle joints (×40, scale bar: 100 μm). **(F)** Relative expression of GPX4 protein in ankle joints (×40, scale bar: 200 μm). Data are expressed as mean ± standard error and analyzed by one-way ANOVA. **p < 0.01 compared with the blank group; #*p* < 0.05 compared with the model group; ##*p* < 0.01 compared with the model group.

Compared with the Ctrl group, the CIA group exhibited significantly increased ankle swelling, thymus index, spleen index, inflammatory cytokine levels, and GPX4 protein expression in ankle joints (*p* < 0.01), confirming successful model establishment. The severity of ankle swelling in the model group gradually increased over time (Figure 10A). On day 7 of treatment, ankle swelling was significantly reduced in the MTX, SCW-L, SCW-M, SCW-H, HZZCW-M, and HZZCW-H groups (*p* < 0.01, *p* < 0.05), while no significant inhibition was observed in the remaining groups (*p* > 0.05). Among the HZZCW groups, the medium dose exhibited the strongest effect, with the efficacy ranking HZZCW-L < HZZCW-H < HZZCW-M. By day 14, ankle swelling was significantly reduced in all treatment groups (*p* < 0.01). Comparison between equal-dose groups showed that swelling in the HZZCW-H group was lower than that in the SCW-H group, whereas swelling in the SCW-L group was lower than that in the HZZCW-L group (*p* < 0.05). Intergroup comparisons revealed that the SCW-L group exhibited lower levels of swelling than the SCW-H group (*p* < 0.05), and the HZZCW-M and HZZCW-H groups displayed lower swelling degrees than the HZZCW-L group (*p* < 0.05). On day 21, all treatment groups significantly reduced ankle swelling (*p* < 0.01). No significant differences were observed among the SCW or HZZCW dose groups. However, swelling in the HZZCW-L group was lower than that in the SCW-M group (*p* < 0.05). The thymus index was significantly reduced in the MTX, SCW-M, HZZCW-M, and HZZCW-H groups (*p* < 0.01). Intergroup comparisons showed that the SCW-M group was lower than the SCW-H group, and the HZZCW-H group was lower than the SCW-H group (*p* < 0.01) (Figure 10B). Similarly, the spleen index was significantly decreased in the MTX, SCW-L, SCW-M, HZZCW-M, and HZZCW-H groups (*p* < 0.01, *p* < 0.05). Intergroup comparisons showed that the SCW-L and SCW-M groups had lower spleen indices than the SCW-H group, while the HZZCW-M and HZZCW-H groups showed lower values than the SCW-H group (*p* < 0.05) (Figure 10B). These results demonstrate that both SCW and HZZCW can alleviate ankle swelling and reduce thymus and spleen indices in CIA model rats.

The ELISA results revealed that the expression levels of IL-6, IL-17, and TNF-α were significantly decreased across all groups (*p* < 0.01, *p* < 0.05) (Figure 10C). Comparison between equal-dose groups showed that HZZCW subgroups exhibited significantly lower inflammatory factor levels than the SCW subgroups (*p* < 0.01, *p* < 0.05). H&E staining results (Figure 10D) demonstrated that the Ctrl group maintained normal ankle structure, intact cartilage surface, normal joint space, and absence of synovial hyperplasia or inflammatory cell infiltration. In contrast, the CIA group exhibited narrowed joint space, damaged cartilage surface, and inflammatory cell infiltration. Administration of SCW or HZZCW improved synovial tissue morphology and reduced inflammatory cell infiltration to varying degrees. Masson staining (Figure 10E). In the Ctrl group, collagen fibers and cartilage tissue were uniformly stained, with a clear boundary between cartilage and subchondral bone. The CIA group displayed disordered collagen fiber arrangement, destruction of cartilage and subchondral bone with blurred boundaries, and inflammatory infiltration. Compared with the CIA group, treatment with SCW or HZZCW restored collagen fiber regularity, improved cartilage integrity, and reduced lesion severity. IHC analysis of ankle joints showed that all groups except SCW-L significantly reduced GPX4 protein expression compared with the CIA group. Intergroup comparison indicated that the HZZCW-L group exhibited lower GPX4 expression than the SCW-L group (*p* < 0.05) (Figure 10F). Collectively, these results demonstrate that both SCW and HZZCW exert anti-inflammatory effects in CIA model rats, with HZZCW exhibiting superior efficacy to SCW.

## 4 Discussion

*Aconitum kusnezoffii* primarily affects pyrimidine metabolism, as well as phenylalanine and tryptophan metabolism. Pyrimidine metabolism plays a central role in the effector functions of immune cells. Among pyrimidine metabolites, uridine is a key nucleoside involved in *de novo* pyrimidine synthesis and essential for maintaining fundamental cellular functions and survival. Uridine has been reported to exert therapeutic effects in metabolic disorders (such as diabetes and obesity), rheumatoid arthritis, acute myocardial infarction, and related complications (Song et al., 2025). In this study, fecal uridine levels were elevated, which may reflect a mild impairment of intestinal absorption.

Phenylalanine metabolism emerged as a common pathway affected by both SCW and HZZCW interventions. Dysregulation of this pathway was associated with pathological conditions such as stroke and heart failure (Dong et al., 2025), and liver injury can further compromise the enzymatic activity required for phenylalanine metabolism (Wang, 2001). Normally, phenylalanine is converted into tyrosine via phenylalanine hydroxylase (PAH), while a minor portion undergoes deamination to form phenylpyruvic acid and phenylacetic acid. Impaired PAH activity results in phenylalanine accumulation and elevated phenylpyruvic acid. Phenylpyruvic acid can be further metabolized into phenylacetic acid, thus increasing phenylacetic acid levels (Strasser et al., 2017). Previous studies have shown decreased fecal phenylalanine levels in patients with chronic diarrhea and diabetes (Guo et al., 2024; Wang et al., 2025). In this study, administration of SCW and HZZCW disrupted fecal phenylalanine metabolism, which may underlie observed toxic symptoms, such as diarrhea. This indicates that phenylalanine metabolism represents a common target of the two interventions.

The porphyrin metabolism pathway was identified as a differential pathway in the feces of SCW and HZZCW. Biliverdin (BV), also referred to as dehydrobilirubin, exists as isomers BVIXα, BVIXβ, BVIXγ, and BVIXδ. BV is a tetrapyrrole pigment generated during heme catabolism (O'Carra and Colleran, 1970). As a metabolite of the biliverdin-bilirubin cycle, biliverdin protects lipids from reactive oxygen species (ROS)-induced damage. It also possesses anti-inflammatory properties, contributes to antiviral drug development, and is used therapeutically in conditions such as lung transplantation injury and hepatic ischemia–reperfusion injury (Yan et al., 2022). HZZCW may exert its detoxification effect by modulating porphyrin metabolism.

Tryptophan metabolism was particularly affected by SCW administration. Tryptophan catabolites are absorbed through the intestinal epithelium and enter systemic circulation, where specific metabolites such as IPA, IE, and IAA exhibit antioxidant and anti-inflammatory effects. Indole also stimulates glucagon-like peptide (GLP-1) release from enteroendocrine cells. GLP-1 can suppress appetite, enhance insulin secretion, and slow gastric emptying. In the liver, indole is metabolized to indoxyl sulfate (IS) via CYP2E1 and sulfotransferase (SULT), which can be cytotoxic at elevated concentrations (Roager and Licht, 2018). SCW administration reduced the intestinal absorption of tryptophan metabolites, leading to their accumulation in feces. In contrast, HZZCW regulated fecal tryptophan metabolism, representing a potential mechanism for the “detoxification while preserving efficacy” effect. Furthermore, the synergistic modulation of the alanine-aspartate–glutamate metabolism and porphyrin metabolism pathways by HZZCW may further enhance its detoxification effect.

Ubiquinone (coenzyme Q10, CoQ10) is a lipophilic molecule involved in cellular signal transduction and substance transport. It inhibits lipid and protein peroxidation, supports cell viability by protecting genetic integrity, and reduces cellular necrosis and apoptosis (Bhagavan and Chopra, 2006; Rauchová et al., 1995; Alleva et al., 2001). In addition, as a key electron and proton carrier in mitochondrial aerobic respiration, adequate ubiquinone is essential for ATP synthesis, and its deficiency has been implicated in various metabolism-related disorders (Aaseth et al., 2021; Hernández-Camacho et al., 2018). Although ubiquinone functions as a classical biological antioxidant, excessive intake may paradoxically exert pro-oxidant effects. Previous studies have reported hemorrhagic effects in normal rats following high-dose ubiquinone supplementation (Takahashi, 1995). In this study, compared with the SCW control group, intervention with HZZCW increased the level of 4-hydroxyphenylpyruvate while decreasing the content of 4-hydroxybenzoic acid in the ubiquinone and other terpenoid-quinone biosynthesis pathways in normal rats. These results suggest that HZZCW may suppress the conversion of 4-hydroxyphenylpyruvate to 4-hydroxybenzoic acid and moderately downregulate basal ubiquinone synthesis, thereby preventing oxidative stress associated with excessive endogenous ubiquinone activity. Meanwhile, the accumulation of 4-hydroxyphenylpyruvate may contribute to sustained antioxidant effects, reduce the accumulation of toxic metabolic byproducts, and modulate tyrosine metabolism, thereby alleviating basal metabolic oxidative burden. Collectively, these findings suggest that HZZCW may exert detoxification effects at the level of endogenous metabolic regulation.

Alterations in the intestinal microbiota represent a crucial mediating pathway of *A. kusnezoffii* processed with *Terminalia chebula* decoction. In this study, *Firmicutes*, *Actinobacteria*, *Bacteroidota*, *Proteobacteria*, and *Verrucomicrobiota* were the dominant intestinal phyla across all groups, accounting for over 90% of total flora. Shifts in the abundance of specific phyla and genera were directly associated with toxic effects. Desulfobacter is mainly associated with intestinal inflammation and metabolic diseases (Kushkevych et al., 2021; Birg and Lin, 2025). *Verrucomicrobiota* and *Deferribacterota* are also associated with inflammatory responses (Wang Y. H. et al., 2024; Bai et al., 2022). At the genus level, *Prevotella* can exacerbate periodontitis-related inflammation and contribute to atherosclerosis (Chen et al., 2025; Xu et al., 2023), suggesting that its correlation with the toxic components of *A. kusnezoffii* may mediate toxicity. *Corynebacterium*, an aerobic, non-spore-forming, Gram-positive bacillus, is typically part of the normal skin and mucosal flora but can act as a pathogen under certain conditions (Renom et al., 2007; Sunn et al., 2018). *Xanthomonas*, a Gram-negative genus of Gammaproteobacteria, comprises more than 35 species and can infect over 400 different plant hosts. It produces type III effector molecules as its primary virulence factor (Timilsina et al., 2024). The synthesis of coproporphyrin III has been reported to depend on *Corynebacterium diphtheriae* (Alves et al., 2024). These findings indicate that HZZCW may reduce the production of toxic metabolites by downregulating *Corynebacterium* and *Xanthomona*s. The increased relative abundance of *Xanthomonas* may reflect the effects of the botanical components themselves, warranting further investigation.

Spearman correlation analysis revealed a close correlation between key gut microbiota and differential fecal metabolites. *Corynebacterium* and *Xanthomonas* were positively correlated with highly toxic prototype components of *Aconitum kusnezoffii*, such as kusnezoline, mesaconitine, and fuziline, while negatively correlated with low-toxicity prototype constituents and metabolites, including benzoylhypaconine and benzoylaconine. These results indicated that the abnormal proliferation of *Corynebacterium* and *Xanthomonas* could promote the accumulation of toxic components of *Aconitum kusnezoffii* and inhibit their metabolic detoxification in feces. *Desulfovibrio* was negatively correlated with mesaconitine and fuziline and positively correlated with 3-Methylxanthine, suggesting that it could facilitate the metabolism of toxic components of *Aconitum kusnezoffii* and reduce toxicity. *Prevotella* was negatively correlated with toxic metabolites such as fuziline and positively correlated with toxic metabolic derivatives, indicating that it could enhance the detoxification and metabolism of toxic constituents. The above correlations demonstrated that *Aconitum kusnezoffii* processed with Chebulae Fructus decoction could regulate the structure of gut microbiota, alter the expression levels of differential fecal metabolites (including prototype components), promote the metabolism of toxic *Aconitum* components by gut microbiota, reduce toxic accumulation, and alleviate oxidative and tissue damage. As an important mechanism underlying the detoxification of processed *Aconitum kusnezoffii*, the interaction between gut microbiota and fecal metabolites achieves the coordinated regulation of the intestinal microenvironment and host metabolism, thereby exerting an overall detoxification effect.

Metabolically, uridine activates mitochondrial ATP-dependent potassium channels (mitoKATP), inducing mitochondrial ROS production (Bulion et al., 2019). *Corynebacterium glutamicum* participates in the biosynthesis of uridine diphosphate glucuronate (UDP-GlcA) and uridine diphosphate *N*-acetylglucosamine (UDP-GlcNAc) (Hu et al., 2025). AvrAC, a type III effector of *Xanthomonas campestris*, functions as a uridylyltransferase (Feng et al., 2012). It can add uridine 5′-monophosphate to the activation loops of BIK1 and RIPK, mask phosphorylation sites, and reduce their kinase activity, thereby suppressing downstream signaling. Main alkaloids in SCW and HZZCW, including Mesaconitine, Fuziline, Beiwutine, Benzoylhypaconine, Benzoylaconine, and Benzoylmesaconine, act as both active and toxic components. Processing *A. kusnezoffii* with *Terminalia chebula* significantly reduces its toxicity, likely via the hydrolysis of diester-type aconitines and the formation of insoluble complexes with tannins (Huang et al., 2022).

At the molecular level, quantitative analyses of gene and protein expression comprehensively characterized the activation profile of the EGFR/PI3K/AKT pathway, further demonstrating the distinctive regulatory advantages of HZZCW. The mRNA expression, total protein, and phosphorylation levels of key molecules in this pathway were simultaneously evaluated. Compared with the Ctrl group, the mRNA and total protein expression levels of EGFR, PI3K, and AKT were significantly increased in both the SCW and HZZCW groups (*p* < 0.01). Notably, the expression levels of these molecules were markedly higher in the HZZCW group than in the SCW group. The phosphorylation ratios of p-AKT/AKT, p-EGFR/EGFR, and p-PI3K/PI3K were significantly elevated in both treatment groups compared with the Ctrl group (p < 0.01), confirming that both raw Aconiti Radix (SCW) and Aconiti Radix processed with Chebulae Fructus decoction (HZZCW) activate the EGFR-dependent PI3K/AKT cascade. Notably, the p-PI3K/PI3K ratio was significantly lower than that observed in the SCW group (*p* < 0.05). This finding suggests that processing with Chebulae Fructus decoction does not suppress the entire pathway; rather, it selectively attenuates excessive PI3K phosphorylation, thereby maintaining a balanced activation state. Collectively, these results provide molecular evidence supporting the regulatory effect of HZZCW on the PI3K/AKT signaling pathway.

The PI3K/Akt pathway is a central regulator of cell survival, oxidative stress responses, and apoptosis through its modulation of downstream Bcl-2 family proteins (Li A. et al., 2022; Li S. et al., 2022). As a key downstream effector of PI3K, AKT phosphorylates multiple target proteins, including mTOR (Guo et al., 2015). Glutamic acid and porphyrin metabolic pathways act as critical upstream modulators of PI3K/AKT pathway activation. Glutamine regulates the PI3K/Akt/mTOR signaling axis and thereby influences cellular proliferation and survival (Alzahrani, 2019). Conversely, excessive glutamate accumulation can bind to NR2B receptors, leading to sustained hyperactivation of the PI3K/Akt pathway (Xie et al., 2024). Biliverdin, a key intermediate product of porphyrin metabolism, also exerts protective effects by activating the PI3K/AKT pathway and mitigating cellular injury (Li, 2013).

Intervention with SCW and HZZCW markedly remodeled the fecal metabolic landscape, particularly affecting glutamate, phenylalanine, and porphyrin metabolism. Alterations in these key metabolites may serve as upstream regulatory signals that influence the transcriptional and translational expression of components within the EGFR/PI3K/AKT signaling pathway. KEGG functional enrichment analysis revealed significant enrichment of protein kinase–related functions among the differential microbial communities. Notably, *Corynebacterium* exhibited a strong positive correlation with the enrichment of protein kinase activity, suggesting that the proliferation of *Corynebacterium* following SCW administration may be a critical microbial driver of kinase-related signaling activation. In contrast, treatment with *Aconitum kusnezoffii* processed using Chebulae Fructus decoction (HZZCW) significantly reduced the abundance of *Corynebacterium*, thereby attenuating excessive activation of this pathway. This observation is consistent with the cascade activation signature of the PI3K/AKT kinase pathway. *Corynebacterium* is identified as one of the core differential genera. An aberrant increase in *Corynebacterium* abundance has also been implicated in the regulation of PI3K/AKT signaling homeostasis (Fleeman, 2023). Lipoteichoic acid derived from the cell wall of *Corynebacterium* can activate TLR2-mediated signaling, inducing PI3K phosphorylation and promoting the release of pro-inflammatory cytokines, ultimately contributing to sustained hyperactivation of the PI3K/AKT pathway (Altonsy et al., 2020; Kao et al., 2005). Therefore, the abnormal enrichment of *Corynebacterium* induced by SCW may represent a microbiota-derived mechanism underlying the elevated p-PI3K/PI3K ratio and enhanced toxicity observed in this group. Conversely, the reduction of *Corynebacterium* abundance following HZZCW treatment may contribute to the suppression of excessive PI3K phosphorylation and the restoration of signaling homeostasis.

These findings are further supported by previous studies demonstrating that raw *Aconitum kusnezoffii* (SCW) can elevate MDA levels, disrupt myocardial fiber architecture, and induce myocardial injury (Zhang et al., 2017). In contrast, benzoylaconine (BAC), a major bioactive product generated during processing, has been reported to exert cardioprotective effects through targeted activation of the PI3K/Akt pathway, inhibition of cardiomyocyte apoptosis, and regulation of autophagic homeostasis (Zhou et al., 2021; Zhou et al., 2025). Taken together, the integrated multi-omics analyses and molecular validation results indicate that HZZCW exerts a bidirectional regulatory effect on the PI3K/AKT signaling network. On the one hand, HZZCW elevates the expression of EGFR, PI3K, and AKT, maintaining an adequate signaling reserve and preserving the cardioprotective functions of the PI3K/AKT pathway. On the other hand, HZZCW reshapes the intestinal microbiota composition and corrects disturbances in glutamate- and porphyrin-related metabolism. These coordinated effects mitigate aberrant hyperphosphorylation, suppress excessive inflammatory signaling, and ultimately alleviate the cardiotoxicity associated with dysregulated PI3K/AKT pathway activation.

Moreover, both SCW and HZZCW alleviated symptoms in the CIA model, confirming their anti-inflammatory efficacy. Under pathological conditions such as rheumatoid arthritis (RA), pro-inflammatory cytokines (IL-1β, IL-6, and IL-8) produced by synovial tissue disrupt the differentiation of osteoclasts and osteoblasts, thereby impairing bone formation (Li et al., 2021). Ferroptosis, a recently characterized form of programmed cell death, is driven by iron-dependent lipid peroxidation and has been implicated in RA pathogenesis through dysregulation of iron metabolism, GSH metabolism, GPX4 activity, and lipid peroxidation (Dixon and Olzmann, 2024). GPX4, a critical peroxidase in ferroptosis pathways (System Xc-/GSH/GPX4 and p53/SLC7A11/GPX4), is the sole intracellular glutathione peroxidase capable of reducing lipid peroxides (Basit et al., 2017). Previous studies have reported decreased GPX4 protein and mRNA levels in peripheral blood mononuclear cells of RA patients (Liu et al., 2024). Zhao et al. (2024) observed elevated GPX4 and SLC7A11 expression in RA fibroblast-like synoviocytes (FLSs) compared to trauma controls. Additionally, TNF-α can modulate the System Xc-/GSH/GPX4 axis via NF-κB activation, thereby protecting synovial fibroblasts (Wu et al., 2022). In this study, both SCW and HZZCW reduced IL-6, IL-17, and TNF-α levels, with HZZCW exhibiting greater efficacy. Pathological assessment revealed improved synovial structure and reduced inflammatory infiltration. GPX4 protein expression was also decreased in all treatment groups except SCW-L, with HZZCW-L showing a more pronounced reduction than SCW-L.

In this study, ankle swelling and serum pro-inflammatory cytokine levels exhibited an inconsistent dose–response relationship, reflecting the distinct biological significance of these two outcome measures. Serum cytokine concentrations assessed at the end of administration primarily represent the systemic inflammatory status, whereas ankle swelling reflects a composite pathological phenotype influenced by local inflammation, vascular permeability, tissue edema, and drug-related physiological responses. Notably, the high-dose raw *A. kusnezoffii* group (SCW-H) demonstrated significantly greater suppression of pro-inflammatory cytokines than the low-dose group (SCW-L), confirming superior anti-inflammatory activity at the molecular level.

However, on day 14 of intervention, SCW-L produced a more pronounced reduction in ankle swelling than SCW-H. This phenomenon does not necessarily imply weaker anti-inflammatory efficacy of SCW-H; rather, it suggests that the gross pathological manifestation of joint swelling may be influenced by factors beyond cytokine inhibition alone. Raw *A. kusnezoffii* contains diester-diterpenoid alkaloids, which are known to exhibit dose-dependent systemic toxicity. At lower doses, the anti-inflammatory effects may predominate, leading to rapid attenuation of synovial inflammation and tissue edema. In contrast, although SCW-H more effectively suppresses inflammatory mediator production, the higher alkaloid burden may simultaneously induce systemic stress responses or vascular dysfunction, which could partially offset the early improvement in joint swelling. By day 21, the superior anti-inflammatory activity of SCW-H became more evident, and the difference in ankle swelling between the two dose groups was substantially diminished. The observed discrepancy may also be related to disease stage. During relatively mild or moderate phases of disease progression, lower doses may be sufficient to control inflammatory manifestations, whereas higher doses may become advantageous as pathological severity increases and stronger suppression of inflammatory signaling is required.

In summary, ankle swelling should be interpreted as an integrated phenotypic outcome rather than a direct surrogate marker of anti-inflammatory potency. While the anti-inflammatory activity of raw *A. kusnezoffii* increases in a dose-dependent manner, systemic toxicity also rises concurrently. This dual dose-dependent pattern of efficacy and toxicity highlights the therapeutic limitations of raw Aconitum kusnezoffii and further supports the rationale for processing with Chebulae Fructus decoction.

Collectively, these findings demonstrated that SCW and HZZCW can effectively alleviate synovial hyperplasia, inflammatory infiltration, and joint tissue pathology in CIA rats, likely via modulation of GPX4-related signaling pathways, inhibition of an overactive antioxidant system, and restoration of synovial sensitivity to ferroptosis.

## 5 Conclusion

This study verifies that *T. chebula* decoction-processed *A. kusnezoffii* exerts pharmacological effects through multiple paths: regulating key metabolic pathways, such as the phenylalanine metabolic pathway; downregulating the intestinal microbiota, notably *Corynebacterium* and *Xanthomonas*; activating the PI3K/Akt pathway; reducing pro-inflammatory cytokines and GPX4 protein expression; and improving the pathological changes in the heart and ankle joints. This study provides a scientific foundation for the safe clinical application of *A. kusnezoffii.*

## Data Availability

Raw bacterial 16S rRNA gene sequencing data derived from gut microbiota in this study have been deposited in the NCBI BioProject database with the accession number PRJNA1481889 (https://www.ncbi.nlm.nih.gov/bioproject/PRJNA1481889). Meanwhile, the corresponding metabolomics raw data have been submitted to the MetaboLights database under the accession number MTBLS14867 (https://www.ebi.ac.uk/metabolights/MTBLS14867).

## References

[ref1] Aaseth J. Alexander J. Alehagen U. (2021). Coenzyme Q(10) supplementation – in ageing and disease. Mech. Ageing Dev. 197:111521. doi: 10.1016/j.mad.2021.111521, 34129891

[ref2] Alleva R. Tomasetti M. Andera L. Gellert N. Borghi B. Weber C. . (2001). Coenzyme Q blocks biochemical but not receptor-mediated apoptosis by increasing mitochondrial antioxidant protection. FEBS Lett. 503, 46–50. doi: 10.1016/S0014-5793(01)02694-1, 11513852

[ref3] Altonsy M. O. Kurwa H. A. Lauzon G. J. Amrein M. Gerber A. N. Almishri W. . (2020). *Corynebacterium tuberculostearicum*, a human skin colonizer, induces the canonical nuclear factor-κB inflammatory signaling pathway in human skin cells. Immunity Inflamm. Dis. 8, 62–79. doi: 10.1002/iid3.284, 31912662 PMC7016847

[ref4] Alves G. B. da Costa Marques Calderari M. R. da Fonseca E. N. Dos Santos L. S. de Mattos-Guaraldi A. L. (2024). Porphyrin production by *Corynebacterium diphtheriae* strains from clinical isolates. Chem. Biodivers. 21:e202401011. 39110090 10.1002/cbdv.202401011

[ref5] Alzahrani A. S. (2019). PI3K/Akt/mTOR inhibitors in cancer: at the bench and bedside. Semin. Cancer Biol. 59, 125–132. doi: 10.1016/j.semcancer.2019.07.009, 31323288

[ref6] Bai X. C. Deng H. Xiao C. L. (2022). Analysis of respiratory microbiota changes in asthmatic mice based on high-throughput sequencing. Chin. J. Microecol. 34, 532–535.

[ref7] Basit F. van Oppen L. M. Schöckel L. Bossenbroek H. M. van Emst-de Vries S. E. Hermeling J. C. . (2017). Mitochondrial complex I inhibition triggers a mitophagy-dependent ROS increase leading to necroptosis and ferroptosis in melanoma cells. Cell Death Dis. 8:e2716. doi: 10.1038/cddis.2017.133, 28358377 PMC5386536

[ref8] Bhagavan H. N. Chopra R. K. (2006). Coenzyme Q10: absorption, tissue uptake, metabolism and pharmacokinetics. Free Radic. Res. 40, 445–453. doi: 10.1080/10715760600617843, 16551570

[ref9] Birg A. Lin H. C. (2025). The role of Bacteria-derived hydrogen sulfide in multiple axes of disease. Int. J. Mol. Sci. 26:3340. doi: 10.3390/ijms26073340, 40244174 PMC11990059

[ref10] Bulion V. V. Selina E. N. Krylova I. B. (2019). Protective effect of uridine on metabolic processes in rat myocardum during its ischemia/reperfusion damage. Biomed Khim 65, 398–402. doi: 10.18097/pbmc20196505398, 31666412

[ref11] Chen B. Bai Y. Tong F. Yan J. Zhang R. Zhong Y. . (2023). Glycoursodeoxycholic acid regulates bile acids level and alters gut microbiota and glycolipid metabolism to attenuate diabetes. Gut Microbes 15:2192155. doi: 10.1080/19490976.2023.2192155, 36967529 PMC10054359

[ref12] Chen Q. Xia T. T. Zhou Y. Q. Chang M. Y. Hu N. Yang Y. M. . (2025). *Prevotella melaninogenica* exacerbates periodontal inflammation and impairs cognitive function in mic. J. South. Med. Univ. 45, 453–460. 10.12122/j.issn.1673-4254.2025.03.02 PMC11955903 40159959

[ref13] Dixon S. J. Olzmann J. A. (2024). The cell biology of ferroptosis. Nat. Rev. Mol. Cell Biol. 25, 424–442. doi: 10.1038/s41580-024-00703-5, 38366038 PMC12187608

[ref14] Dong Z. Y. Zhu W. H. Sun X. Y. Ying Z. H. Pu X. Y. Guo D. Q. . (2025). Metabolomic effects of Zhongfengting granules on rats with acute cerebral ischemia model. World Sci. Technol. Mod. Tradit. Chin. Med. 27, 564–572.

[ref15] Feng F. Yang F. Rong W. Wu X. Zhang J. Chen S. . (2012). A Xanthomonas uridine 5′-monophosphate transferase inhibits plant immune kinases. Nature 485, 114–118. doi: 10.1038/nature10962, 22504181

[ref16] Fleeman R. (2023). Repurposing inhibitors of phosphoinositide 3-kinase as adjuvant therapeutics for bacterial infections. Front Antibiot 2:1135485. doi: 10.3389/frabi.2023.1135485, 38983593 PMC11233138

[ref17] Guo H. German P. Bai S. Barnes S. Guo W. Qi X. . (2015). The PI3K/AKT pathway and renal cell carcinoma. J. Genet. Genomics 42, 343–353. doi: 10.1016/j.jgg.2015.03.003, 26233890 PMC4624215

[ref18] Guo S. Ma T. Kwok L. Y. Quan K. Li B. Wang H. . (2024). Effects of postbiotics on chronic diarrhea in young adults: a randomized, double-blind, placebo-controlled crossover trial assessing clinical symptoms, gut microbiota, and metabolite profiles. Gut Microbes 16:2395092. doi: 10.1080/19490976.2024.2395092, 39189588 PMC11352714

[ref19] Gupta A. Kumar R. Bhattacharyya P. Bishayee A. Pandey A. K. (2020). *Terminalia bellirica* (Gaertn.) roxb. (Bahera) in health and disease: a systematic and comprehensive review. Phytomedicine 77:153278. doi: 10.1016/j.phymed.2020.153278, 32781393

[ref20] Hernández-Camacho J. D. Bernier M. López-Lluch G. Navas P. (2018). Coenzyme Q(10) supplementation in aging and disease. Front. Physiol. 9:44. doi: 10.3389/fphys.2018.00044, 29459830 PMC5807419

[ref21] Hu L. Xiao S. Sun J. Wang F. Yin G. Xu W. . (2025). Regulating cellular metabolism and morphology to achieve high-yield synthesis of hyaluronan with controllable molecular weights. Nat. Commun. 16:2076. doi: 10.1038/s41467-025-56950-3, 40021631 PMC11871322

[ref22] Huang H. Chen T. F. Li H. Li L. Gao Y. H. Song L. . (2022). Pharmacokinetic study of aconitum alkaloids in rats determined by liquid chromatography-mass spectrometry (LC-MS). World J. Tradit. Chin. Med. 17, 3472–75+80.

[ref23] Kang H. Q. Li H. L. Li W. Q. Yang C. H. Cui X. Y. He P. X. . (2025). To explore the mechanism of Yiqi Juanbi formula in the treatment of rheumatoid arthritis synovitis based on the JAK2/STAT3. Chin. J. Osteoporos. 31, 1099–1106.

[ref24] Kao S. J. Lei H. C. Kuo C. T. Chang M. S. Chen B. C. Chang Y. C. . (2005). Lipoteichoic acid induces nuclear factor-kappaB activation and nitric oxide synthase expression via phosphatidylinositol 3-kinase, Akt, and p38 MAPK in RAW 264.7 macrophages. Immunology 115, 366–374. doi: 10.1111/j.1365-2567.2005.02160.x, 15946254 PMC1782163

[ref25] Kushkevych I. Dordević D. Vítězová M. (2021). Possible synergy effect of hydrogen sulfide and acetate produced by sulfate-reducing bacteria on inflammatory bowel disease development. J. Adv. Res. 27, 71–78. doi: 10.1016/j.jare.2020.03.007, 33318867 PMC7728581

[ref26] Li W. (2013). Study on the Inhibitory Effect and Mechanism of Biliverdin against Hydrogen Peroxide-Induced Apoptosis in Rat Proximal Renal Tubular Epithelial Cells, Master. Wuhan: Huazhong University of Science and Technology.

[ref27] Li Y. Liu R. H. Huang S. B. Zou M. Li L. M. He H. W. (2021). Research progress on the pathogenesis and therapeutic drugs of rheumatoid arthritis. China Dispens. 32, 2154–2159.

[ref28] Li S. Yu L. Shi Q. Liu Y. Zhang Y. Wang S. . (2022). An insight into current advances on pharmacology, pharmacokinetics, toxicity and detoxification of aconitine. Biomed. Pharmacother. 151:113115. doi: 10.1016/j.biopha.2022.113115, 35605296

[ref29] Li A. Zhao F. Yang T. Zhao Y. Liu H. Yang S. . (2022). PTX3/TWIST1 feedback loop modulates lipopolysaccharide-induced inflammation via PI3K/Akt signaling pathway. J. Interf. Cytokine Res. 42, 161–169. doi: 10.1089/jir.2021.0183, 35438530

[ref30] Li X. R. Zhong J. W. Gao T. Jiang B. Ma X. H. (2025). Study on the regulatory effect of Liuwei Dihuang pills on the intestinal flora of aging rats. Liaoning J. Tradit. Chin. Med. 52, 187–92+228-29.

[ref31] Liu C. Ma W. K. Chen C. M. An Y. Jiang Z. Huang H. (2024). Expression of the system xc-/GSH/GPX4 ferroptosis pathway in peripheral blood mononuclear cells of patients with rheumatoid arthritis and its impact on inflammatory cytokine secretion. J. Anhui Med. Univ. 59, 64–70.

[ref32] O'Carra P. Colleran E. (1970). Separation and identification of biliverdin isomers and isomer analysis of phycobilins and bilirubin. J. Chromatogr. 50, 458–468. doi: 10.1016/s0021-9673(00)97973-1, 5449461

[ref33] Prasad S. Srivastava S. K. (2020). Oxidative stress and cancer: chemopreventive and therapeutic role of Triphala. Antioxidants 9:72. doi: 10.3390/antiox9010072, 31941067 PMC7022920

[ref34] Rauchová H. Drahota Z. Lenaz G. (1995). Function of coenzyme Q in the cell: some biochemical and physiological properties. Physiol. Res. 44, 209–216. 8789639

[ref35] Renom F. Garau M. Rubí M. Ramis F. Galmés A. Soriano J. B. (2007). Nosocomial outbreak of *Corynebacterium striatum* infection in patients with chronic obstructive pulmonary disease. J. Clin. Microbiol. 45, 2064–2067. doi: 10.1128/JCM.00152-07, 17409213 PMC1933039

[ref36] Roager H. M. Licht T. R. (2018). Microbial tryptophan catabolites in health and disease. Nat. Commun. 9:3294. doi: 10.1038/s41467-018-05470-4, 30120222 PMC6098093

[ref37] Rong B. S. Huang K. L. Yuan Y. L. Wang J. H. Li X. Ma C. J. (2021). Research progress on chemical constituents and pharmacological effects of *Aconitum* medicinal materials. Chin. Pharm. Aff. 35, 932–947.

[ref38] Song C. Liu Z. J. Xu B. Zou R. Hu W. (2025). The role of uridine in health and disease. J. Inflamm. Res. 18, 10163–10179. doi: 10.2147/JIR.S506308, 40761385 PMC12318844

[ref39] Strasser B. Sperner-Unterweger B. Fuchs D. Gostner J. M. (2017). Mechanisms of inflammation-associated depression: immune influences on tryptophan and phenylalanine metabolisms. Curr. Top. Behav. Neurosci. 31, 95–115. doi: 10.1007/7854_2016_23, 27278641

[ref40] Sunn W. Li Y. N. Su J. R. (2018). Epidemiological characteristics and antimicrobial susceptibility analysis of *Corynebacterium* infections. J. Clin. Exp. Med. 17, 2228–2231.

[ref41] Takahashi O. (1995). Haemorrhagic toxicity of a large dose of alpha-, beta-, gamma- and delta-tocopherols, ubiquinone, beta-carotene, retinol acetate and L-ascorbic acid in the rat. Food Chem. Toxicol. 33, 121–128. doi: 10.1016/0278-6915(94)00120-D, 7867999

[ref42] Timilsina S. Kaur A. Sharma A. Ramamoorthy S. Vallad G. E. Wang N. . (2024). Xanthomonas as a model system for studying pathogen emergence and evolution. Phytopathology 114, 1433–1446. doi: 10.1094/PHYTO-03-24-0084-RVW, 38648116

[ref43] Wang Q. P. (2001). Phenylalanine metabolism disorders and diseases. Foreign Med Sci, 6, 451–453.

[ref44] Wang W. Cao M. Y. Liao W. X. Zhu H. Zhang H. Y. Huang C. Q. X. . (2025). GC-MS-based fecal metabolomics study on the onset and progression of diabetes mellitus in mice and the intervention effects of mulberry leaf combined with metformin. Chin. J. Hosp. Pharm. 45, 634–643.

[ref45] Wang Y. H. Huang N. N. Li B. Chen H. Q. Li X. B. Chen R. (2024). Research progress on the regulation of intestinal bacterial abundance by ambient particulate matter exposure. J. Environ. Occup. Med. 41, 451–456.

[ref46] Wang Z. Y. Shen H. R. Han Y. X. Jiang J. D. Jiandong Jiang W. . (2024). Effects of enzymes produced by intestinal flora and natural medicines on diseases. Acta Pharm. Sin. 59, 2183–2191.

[ref47] Wu J. Feng Z. Chen L. Li Y. Bian H. Geng J. . (2022). TNF antagonist sensitizes synovial fibroblasts to ferroptotic cell death in collagen-induced arthritis mouse models. Nat. Commun. 13:676. doi: 10.1038/s41467-021-27948-4, 35115492 PMC8813949

[ref48] Wu X. Yuan M. M. Xiong X. L. Zhong G. H. (2021). Research progress on chemical constituents and pharmacological effects of *Aconitum kusnezoffii*. Drug Eval 18, 1534–1536.

[ref49] Xie Y. Hu G. Liu H. (2024). Effects of Guipi decoction on glutamate and PI3K/Akt/mTOR signaling pathway in rats with autism spectrum disorder of heart-spleen deficiency pattern. Mod J Integr Tradit Chin West Med 33, 1461–68+77.

[ref50] Xu Z. Lian C. Pan L. Lai W. Zhang F. Peng L. . (2023). N-acetyl-L-leucine protects MPTP-treated Parkinson’s disease mouse models by suppressing Desulfobacterota via the gut-brain axis. Brain Res. Bull. 202:110729. doi: 10.1016/j.brainresbull.2023.110729, 37579888

[ref51] Yan S. H. Shao M. L. Xu M. J. Zhang X. Yang T. W. Rao Z. M. (2022). Efficient synthesis of biliverdin by whole – cell catalysis of recombinant *Escherichia coli*. Chin. J. Biotechnol. 38, 2581–2593. 10.13345/j.cjb.220137 35871626

[ref52] Yang X. N. (2022). Separation, Characterization and In Vivo Metabolite Identification of Chemical Constituents in Panax Notoginseng Flowers Based on High-Resolution Liquid Chromatography-Mass Spectrometry (HR-LC-MS), Master. Tianjin: Tianjin University of Traditional Chinese Medicine.

[ref53] Yuan W. W. Wang H. C. Chen Y. T. Rong Y. G. Chen W. W. Zhao J. X. . (2021). Analysis of intestinal flora structure and co-occurrence network in populations with different body mass indices. Microbiol. Bull. 48, 3776–3790.

[ref54] Zhang X. F. Cui Y. T. Miao X. Liu D. D. Ma Z. X. Li G. (2017). Protective effect of Mongolian medicine Chebula on *Aconitum kusnezoffii*-induced cardiotoxicity in rats. J. Chin. Med. Mater. 40, 2693–2696.

[ref55] Zhang X. F. Song L. Tu Y. Na S. S. Wu L. Q. G. (2016). Comparative study on the contents of three aconitines in Mongolian medicine aconite root from three different habitats before and after processing. J. Chin. Ethnomedicine 22, 42–44.

[ref56] Zhao C. Yu Y. Yin G. Xu C. Wang J. Wang L. . (2024). Sulfasalazine promotes ferroptosis through AKT-ERK1/2 and P53-SLC7A11 in rheumatoid arthritis. Inflammopharmacology 32, 1277–1294. doi: 10.1007/s10787-024-01439-6, 38407703 PMC11006818

[ref57] Zhou C. Gao J. Ji H. Li W. Xing X. Liu D. . (2021). Benzoylaconine modulates LPS-induced responses through inhibition of toll-like receptor-mediated NF-κB and MAPK signaling in RAW264.7 cells. Inflammation 44, 2018–2032. doi: 10.1007/s10753-021-01478-z, 34272638

[ref58] Zhou W. M. Lang S. K. Ge X. Jiang W. Jia D. Jia D. . (2025). Benzoylaconitine alleviates oxygen-glucose deprivation/reoxygenation-induced myocardial cell injury via the phosphatidylinositol 3-kinase/protein kinase B pathway. Chin. J. Geriatr. Heart Brain Vessel Dis. 27, 211–216.

