# Correction: *Terminalia chebula* decoction-processed *Aconitum kusnezoffii* preserves therapeutic effects through regulation of metabolism and intestinal microbiota

**DOI:** 10.3389/fmicb.2026.1958966

**Published:** 2026-08-17

**Authors:** Rina Sa, Li Mei, Gege Chen, Yasila Ba, Yongchang Bao, Liwei Guan, Rigen Te, Riliga Wu, Huricha Baigude, Lin Song

**Affiliations:** 1 College of Mongolian Medicine, Inner Mongolia Medical University, Hohhot, Inner Mongolia, China; 2 Medical Simulation Center, Inner Mongolia Medical University, Hohhot, Inner Mongolia, China; 3 Experimental Center, Inner Mongolia Traditional Chinese & Mongolian Medical Research Institute, Hohhot, Inner Mongolia, China; 4 School of Chemistry & Chemical Engineering, Inner Mongolia University, Hohhot, China; 5 Medical Innovation Center for Nationalities, Inner Mongolia Medical University, Hohhot, Inner Mongolia, China

**Keywords:** *A. kusnezoffii*, intestinal flora, metabolomics, pharmacodynamic effect, PI3K/AKT, *Terminalia chebula* decoction processing

In the published article, the affiliation “Medical Innovation Center for Nationalities, Inner Mongolia Medical University, Hohhot, Inner Mongolia, China” was omitted for author Lin Song. It has been added as affiliation 5, and Lin Song's assigned affiliation has been updated from “3” to “5” in the author list.

The original version of this article has been updated.

