# Supplementary material for: Terminalia chebula decoction-processed Aconitum kusnezoffii preserves therapeutic effects through regulation of metabolism and intestinal microbiota
Source: Front Microbiol. 2026 Aug 4;17:1879939. doi: 10.3389/fmicb.2026.1879939 (PMC13528435; doi:10.3389/fmicb.2026.1879939)
Supplement: Supplementary file 1 [file Table_1.DOCX]

**Supplementary Information**

**Terminalia chebula decoction-processed Aconitum kusnezoffii preserves therapeutic effects through regulation of metabolism and intestinal microbiota**

Rina Sa^,1^, Li Mei^1^, Gege Chen^1^, Yasila Ba^2^, Yongchang Bao^2^, Liwei Guan^1^, Riliga Wu^1^, Rigen Te^1^, Huricha Baigude^3^*, Lin Song^1^*

*1.Medical Innovation Center for Nationalities, Inner Mongolia Medical University, Hohhot, Inner Mongolia, 010110, P.R. China*

*2. Experimental center, Inner Mongolia Traditional Chinese & Mongolian Medical Research Institute, Hohhot, Inner Mongolia, 010017, P.R. China*

*3. School of Chemistry & Chemical Engineering, Inner Mongolia University, Hohhot 010020, China*

Correspondence to:

*Prof. Lin Song: Medical Innovation Center for Nationalities, Inner Mongolia Medical University, Hohhot, Inner Mongolia, 010110, P.R. China. Email: songlinwps@163.com

*Prof. Huricha Baigude: School of Chemistry & Chemical Engineering, Inner Mongolia University, Hohhot, 010020, P.R. China. Email: hbaigude@imu.edu.cn

Supplementary Table 1 Sequences of RT-qPCR primers

| Gene | Forword（5’→3’） | Reverse（5’→3’） |
| --- | --- | --- |
| PI3K | TGTGGCACAGACTTGGTGTT | TTCTTCCCTTGAGATGTCTCCC |
| AKT | TGTCTCGTGAGCGCGTGTT | CCGTTATCTTGATGTGCCCGTC |
| EGFR | TTCTTTCACCCGCACTCCTC | CCGCCAGAGAAAACTGACCA |

**Supplementary Figure 1.** Total ion chromatograms (TICs) in positive and negative ion modes. (A) TICs of fecal samples from the three groups in positive ion mode. (B) TICs of fecal samples from the three groups in negative ion mode.

**Supplementary Figure 2.** Volcano plots of differential metabolites. (A) Volcano plot of differential metabolites in SCW feces. (B) Volcano plot of differential metabolites in HZZCW feces. (C) Volcano plot of differential metabolites in feces between the two groups.

**Supplementary Figure 3.** Bar charts of differential bacterial phyla among the three groups. (A) Bar chart of differential bacterial phyla among the three groups, P < 0.05. (B) Bar chart of differential bacterial phyla between SCW and HZZCW groups, P < 0.05.
