# Supplementary material for: Terminalia chebula decoction-processed Aconitum kusnezoffii preserves therapeutic effects through regulation of metabolism and intestinal microbiota
Source: Front Microbiol. 2026 Aug 4;17:1879939. doi: 10.3389/fmicb.2026.1879939 (PMC13528435; doi:10.3389/fmicb.2026.1879939)
Supplement: Supplementary file 2 [file Table_2.DOCX]

Supplementary Table2-1 Potential differential metabolites of SCW in feces

| No. | MS2 name | mz | ppm （10-6） | rt（min） | MS2 | ms2Adduct | Formula | VIP | P-Value | ctrl vs. SCW |
| --- | --- | --- | --- | --- | --- | --- | --- | --- | --- | --- |
| 1 | Trigonelline | 138.0543 | 4.6 | 2.87 | 138.1;94.1 | [M+H]+ | C7H7NO2 | 2.3273 | 0.0053 | ↑** |
| 2 | Citrulline | 176.1021 | 5 | 3.85 | 159.1;113.1;70.1 | [M+H]+ | C6H13N3O3 | 1.5111 | 0.0022 | ↑** |
| 3 | Pipecolic acid | 130.0857 | 4.7 | 3.10 | 70.1;112.1;71 | [M+H]+ | C6H11NO2 | 1.4146 | 0.0281 | ↑* |
| 4 | 5-methyl-2'-deoxycytidine | 242.1122 | 5.5 | 1.74 | 126.1;194.1;217.1 | [M+H]+ | C10H15N3O4 | 1.3018 | 0.0216 | ↑* |
| 5 | Ectoine | 143.0808 | 5 | 3.35 | 143.1;97.1;101.1 | [M+H]+ | C6H10N2O2 | 4.4794 | 0.0044 | ↑** |
| 6 | Stachydrine | 144.1012 | 5.2 | 2.71 | 144.1 | [M+H]+ | C7H13NO2 | 1.7242 | 0.0105 | ↑* |
| 7 | N2-Acetyllysine | 189.1225 | 5 | 3.83 | 129.1;84.1;189.1 | [M+H]+ | C8H16N2O3 | 1.2987 | 0.0083 | ↑** |
| 8 | trans-Aconitic acid | 173.0078 | 7.7 | 4.19 | 85;129;111 | [M-H]- | C6H6O6 | 1.2334 | 0.0054 | ↑** |
| 9 | 5-Aminopentanoic acid | 118.0857 | 5.1 | 3.81 | 118.1;72.1;101.1 | [M+H]+ | C5H11NO2 | 1.5782 | 0.0468 | ↑* |
| 10 | Creatine | 132.0758 | 7.2 | 3.45 | 132.1;90.1;44 | [M+H]+ | C4H9N3O2 | 1.2152 | 0.0015 | ↓** |
| 11 | Pantothenic acid | 220.1169 | 4.9 | 2.67 | 90.1;220.1;202.1 | [M+H]+ | C9H17NO5 | 1.0874 | 0.0187 | ↓* |
| 12 | Phenylalanine | 166.0854 | 5.6 | 2.63 | 120.1;121.1;166.1 | [M+H]+ | C9H11NO2 | 1.1376 | 0.0040 | ↑** |
| 13 | Nicotinamide | 123.0546 | 5.6 | 0.48 | 123.1;80 | [M+H]+ | C6H6N2O | 1.4907 | 0.0209 | ↑* |
| 14 | S-Adenosylmethionine | 399.1427 | 4.5 | 4.98 | 250.1;136.1;366.2 | [M+H]+ | C15H22N6O5S | 1.2403 | 0.0179 | ↓* |
| 15 | 5-Methylcytosine | 126.0655 | 6 | 1.74 | 126.1;125.1;97.1 | [M+H]+ | C5H7N3O | 1.3290 | 0.0174 | ↑* |
| 16 | 3-Phenylpropanoic acid | 149.0594 | 9 | 0.60 | 149;149.1;59 | [M-H]- | C9H10O2 | 1.5343 | 0.0321 | ↑* |
| 17 | Pyroglutamic acid | 128.0342 | 8.3 | 2.98 | 128 | [M-H]- | C5H7NO3 | 1.2415 | 0.0046 | ↑** |
| 18 | Indolelactic acid | 204.0648 | 8.6 | 0.97 | 59;204.1;62 | [M-H]- | C11H11NO3 | 2.4066 | 0.0032 | ↑** |
| 19 | Indoleacetic acid | 174.0545 | 8.8 | 0.85 | 173.1;127.1;130.1 | [M-H]- | C10H9NO2 | 2.8051 | 0.0051 | ↑** |
| 20 | Vigabatrin | 128.0707 | 7.9 | 3.11 | 128.1;128;85 | [M-H]- | C6H11NO2 | 1.2850 | 0.0130 | ↑* |
| 21 | Pyruvate | 87.008 | 8.7 | 0.84 | 87 | [M-H]- | C3H4O3 | 1.9013 | 0.0039 | ↓** |
| 22 | Erucic acid | 337.3083 | 8.5 | 0.33 | 255.2;337.3;337.2 | [M-H]- | C22H42O2 | 1.3846 | 0.0406 | ↓* |
| 23 | Hydroxyisocaproic acid | 131.0702 | 8.8 | 0.68 | 131.1;85;85.1 | [M-H]- | C6H12O3 | 1.9449 | 0.0073 | ↑** |
| 24 | 3-Indoleacrylic acid | 186.0544 | 8.9 | 0.68 | 142.1;186.1;143.1 | [M-H]- | C11H9NO2 | 2.4083 | 0.0182 | ↑* |
| 25 | Azelaic acid | 187.0962 | 7.3 | 3.40 | 187.1;125.1;159 | [M-H]- | C9H16O4 | 1.1313 | 0.0134 | ↑* |
| 26 | 2'-Deoxyuridine | 227.0653 | 8.8 | 0.70 | 184.1;227.1;94 | [M-H]- | C9H12N2O5 | 1.1841 | 0.0294 | ↑* |
| 27 | 21-Deoxycortisol | 405.2246 | 8.9 | 0.34 | 59;62;405.2 | [M+CH3COO]- | C21H30O4 | 2.1096 | 0.0459 | ↓* |
| 28 | Docosahexaenoic acid (DHA) | 327.2301 | 8.8 | 0.33 | 327.2;283.2;59 | [M-H]- | C22H32O2 | 1.0292 | 0.0366 | ↑* |
| 29 | beta-Guanidinopropionic acid | 130.061 | 8.8 | 3.47 | 88;130.1;128 | [M-H]- | C4H9N3O2 | 1.5329 | 0.0006 | ↓** |
| 30 | Phenylacetylglycine | 194.0801 | 5.7 | 1.61 | 91.1;76;194.1 | [M+H]+ | C10H11NO3 | 1.8941 | 0.0135 | ↑* |
| 31 | 1-Palmitoyllysophosphatidate | 393.2382 | 4.6 | 2.80 | 155;57.1;99 | [M-H2O+H]+ | C19H39O7P | 1.5775 | 0.0126 | ↑* |
| 32 | PC(16:0/18:1(9Z)) | 760.578 | 9.3 | 0.33 | 184.1;89.1;99.4 | [M+H]+ | C42H82NO8P | 1.2053 | 0.0340 | ↑* |
| 33 | 3-Methylcrotonylglycine | 156.0652 | 9 | 1.82 | 155.9;74;59 | [M-H]- | C7H11NO3 | 1.4396 | 0.0011 | ↑** |
| 34 | Dodecanoic acid | 199.1686 | 8.8 | 0.42 | 199.2;59;157 | [M-H]- | C12H24O2 | 1.1503 | 0.0417 | ↑* |
| 35 | Glycolithocholic acid 3-sulfate | 512.2644 | 8.5 | 1.65 | 512.3;56.9 | [M-H]- | C26H43NO7S | 2.1300 | 0.0269 | ↑* |
| 36 | N-Acetylhistamine | 154.0968 | 4.9 | 0.93 | 154.1;95.1;112.1 | [M+H]+ | C7H11N3O | 1.2424 | 0.0367 | ↑* |
| 37 | Imatinib | 492.2539 | 4.5 | 2.16 | 492.3;313;62 | [M-H]- | C29H31N7O | 1.7174 | 0.0330 | ↑* |
| 38 | S1P(d18:0) | 380.2543 | 7.4 | 3.10 | 79;380.3;201 | [M-H]- | C18H40NO5P | 1.6389 | 0.0059 | ↓** |
| 39 | Bilirubin | 585.2682 | 4.4 | 0.44 | 299.1;300.1;585.3 | [M+H]+ | C33H36N4O6 | 1.4500 | 0.0209 | ↑* |
| 40 | DHA ethyl ester | 357.2772 | 4.4 | 0.86 | 357.3;95.1;161.1 | [M+H]+ | C24H36O2 | 2.0947 | 0.0380 | ↑* |
| 41 | Monomethyl fumarate | 129.0182 | 8.4 | 1.61 | 128;128.9;88 | [M-H]- | C5H6O4 | 1.6429 | 0.0041 | ↑** |

Supplementary Table2-2 Potential differential metabolites of HZZCW in feces

| No. | MS2 name | mz | ppm（10-6） | rt（min） | MS2 | ms2Adduct | Formula | VIP | P-Value | Ctrl vs.HZZCW |
| --- | --- | --- | --- | --- | --- | --- | --- | --- | --- | --- |
| 1 | Dimethylglycine | 104.0701 | 5.1 | 3.01 | 58.1;104.1;60.1 | [M+H]+ | C4H9NO2 | 1.4258 | 0.0439 | ↓* |
| 2 | Stachydrine | 144.1012 | 5.2 | 2.71 | 144.1 | [M+H]+ | C7H13NO2 | 1.3798 | 0.0495 | ↑* |
| 3 | Car(18:1) | 426.3559 | 4.5 | 1.56 | 85;426.4;60.1 | [M+H]+ | C25H47NO4 | 1.2037 | 0.0300 | ↓* |
| 4 | Arachidonoylcarnitine (Car(20:4)) | 448.3401 | 4.6 | 1.48 | 448.3;85;60.1 | [M+H]+ | C27H45NO4 | 1.7022 | 0.0084 | ↓** |
| 5 | Kynurenine | 209.091 | 5 | 2.69 | 94.1;192.1;136.1 | [M+H]+ | C10H12N2O3 | 1.4688 | 0.0045 | ↓** |
| 6 | Phenylalanine | 166.0854 | 5.6 | 2.63 | 120.1;121.1;166.1 | [M+H]+ | C9H11NO2 | 1.2498 | 0.0036 | ↑** |
| 7 | 2-Hydroxy-2-methylbutyric acid | 117.0548 | 8 | 1.01 | 116.9;117.1;71 | [M-H]- | C5H10O3 | 1.3316 | 0.0243 | ↑* |
| 8 | Pyroglutamic acid | 128.0342 | 8.3 | 2.98 | 128 | [M-H]- | C5H7NO3 | 1.0162 | 0.0154 | ↑* |
| 9 | Indolelactic acid | 204.0648 | 8.6 | 0.97 | 59;204.1;62 | [M-H]- | C11H11NO3 | 1.5234 | 0.0415 | ↑* |
| 10 | Indoleacetic acid | 174.0545 | 8.8 | 0.85 | 173.1;127.1;130.1 | [M-H]- | C10H9NO2 | 2.1566 | 0.0336 | ↑* |
| 11 | Histamine | 112.0863 | 5.9 | 3.62 | 70.1;112.1;112 | [M+H]+ | C5H9N3 | 1.0829 | 0.0135 | ↓* |
| 12 | Pyruvate | 87.008 | 8.7 | 0.84 | 87 | [M-H]- | C3H4O3 | 1.7467 | 0.0108 | ↓* |
| 13 | 4-(Methylamino)butanoic acid | 116.0707 | 8.2 | 3.47 | 116.1;73;59 | [M-H]- | C5H11NO2 | 2.0598 | 0.0042 | ↑** |
| 14 | Acetylglycine | 116.0344 | 7.9 | 2.77 | 74;116;116 | [M-H]- | C4H7NO3 | 2.2188 | 0.0004 | ↑** |
| 15 | 21-Deoxycortisol | 405.2246 | 8.9 | 0.34 | 59;62;405.2 | [M+CH3COO]- | C21H30O4 | 3.4508 | 0.0443 | ↓* |
| 16 | Arachidonic acid (AA) | 303.2302 | 8.9 | 0.33 | 303.2;259.2;59 | [M-H]- | C20H32O2 | 1.2754 | 0.0331 | ↓* |
| 17 | Heptadecanoic acid | 269.2462 | 8.8 | 0.33 | 269.2;268.2 | [M-H]- | C17H34O2 | 1.1198 | 0.0457 | ↓* |
| 18 | 4-Cresol sulfate | 187.0054 | 8.7 | 0.24 | 187;107;80 | [M-H]- | C7H8O4S | 2.9016 | 0.0484 | ↑* |
| 19 | Myristoylcarnitine | 372.3089 | 5.1 | 1.73 | 85;372.3;60.1 | [M+H]+ | C21H41NO4 | 1.2607 | 0.0356 | ↓* |
| 20 | Octanoylcarnitine (Car(8:0)) | 288.2156 | 4.8 | 2.12 | 85;288.2;229.1 | [M+H]+ | C15H29NO4 | 1.3569 | 0.0235 | ↓* |
| 21 | Phenylacetylglycine | 194.0801 | 5.7 | 1.61 | 91.1;76;194.1 | [M+H]+ | C10H11NO3 | 1.6782 | 0.0314 | ↑* |
| 22 | 13(S)-HODE | 295.2252 | 8.8 | 0.42 | 295.2;222.2;237.1 | [M-H]- | C18H32O3 | 1.2381 | 0.0318 | ↓* |
| 23 | Lauroylcarnitine | 344.2776 | 5.6 | 1.84 | 85;344.3;60.1 | [M+H]+ | C19H37NO4 | 1.1133 | 0.0355 | ↓* |
| 24 | Stearidonic acid | 275.1989 | 9.8 | 0.36 | 275.2;231.2;59 | [M-H]- | C18H28O2 | 2.0044 | 0.0354 | ↓* |
| 25 | 3-Hydroxy-4-aminopyridine | 111.0547 | 5.2 | 2.54 | 111.1;83.1;82.1 | [M+H]+ | C5H6N2O | 1.4068 | 0.0297 | ↑* |
| 26 | 3-Methylcrotonylglycine | 156.0652 | 9 | 1.82 | 155.9;74;59 | [M-H]- | C7H11NO3 | 1.9361 | 0.0037 | ↑** |
| 27 | N-Cinnamoylglycine | 204.0649 | 8.5 | 1.62 | 59;160.1;62 | [M-H]- | C11H11NO3 | 1.6851 | 0.0243 | ↑* |
| 28 | N-Acetylcarnosine | 267.1077 | 7.9 | 3.31 | 154.1;267.1;164.1 | [M-H]- | C11H16N4O4 | 1.7899 | 0.0000 | ↓** |
| 29 | Glu-Arg | 304.1603 | 4 | 4.63 | 304.2;175.1;116.1 | [M+H]+ | C11H21N5O5 | 1.3125 | 0.0162 | ↓* |
| 30 | Orotic acid | 155.0086 | 8.1 | 2.26 | 99.9;111;128 | [M-H]- | C5H4N2O4 | 1.0013 | 0.0482 | ↓* |
| 31 | 3-Methoxy-4-hydroxyphenylglycol sulfate | 263.0205 | 9.8 | 0.40 | 263;165.1;150 | [M-H]- | C9H12O7S | 1.4420 | 0.0006 | ↑** |
| 32 | p-Cresol glucuronide | 283.0799 | 8.5 | 2.23 | 113;107;85 | [M-H]- | C13H16O7 | 3.2633 | 0.0078 | ↑** |
| 33 | 2-Methylhippuric acid | 192.065 | 8.6 | 1.62 | 74;192.1;75 | [M-H]- | C10H11NO3 | 2.0357 | 0.0132 | ↑* |
| 34 | all-trans-5,6-Epoxyretinoic acid | 315.1937 | 8.9 | 0.68 | 315.2;271.2;313.2 | [M-H]- | C20H28O3 | 2.5771 | 0.0227 | ↓* |
| 35 | S1P(d18:0) | 380.2543 | 7.4 | 3.10 | 79;380.3;201 | [M-H]- | C18H40NO5P | 1.5294 | 0.0106 | ↓* |
| 36 | 2,2,6,6-Tetramethyl-4-piperidinyl 2-methylacrylate | 226.179 | 5.3 | 0.73 | 226.2;180.2;163.1 | [M+H]+ | C13H23NO2 | 1.2526 | 0.0331 | ↑* |
| 37 | Inosine | 267.07 | 13.1 | 2.03 | 135;89;267.1 | [M-H]- | C10H12N4O5 | 1.7696 | 0.0076 | ↓** |
| 38 | Diglycine | 131.0452 | 7.8 | 3.73 | 130.1;113;114 | [M-H]- | C4H8N2O3 | 1.0103 | 0.0396 | ↑* |
| 39 | Caffeic acid | 181.0519 | 13.1 | 1.28 | 69;163;99 | [M+H]+ | C9H8O4 | 1.1841 | 0.0201 | ↓* |

Supplementary Table2-3 Potential differential metabolites in feces between the two groups

| No. | MS2 name | mz | ppm | rt | MS2 | ms2Adduct | Formula | VIP | P-Value | SCW vs.HZZCW |
| --- | --- | --- | --- | --- | --- | --- | --- | --- | --- | --- |
| 1 | 4-Hydroxybenzoic acid | 137.0239 | 3.4 | 1.3650 | 93;137;121 | [M-H]- | C7H6O3 | 1.5752 | 0.0219 | ↓* |
| 2 | 2,6-Dihydroxybenzoic acid | 153.0187 | 3.7 | 0.2350 | 153;109;135 | [M-H]- | C7H6O4 | 1.4688 | 0.0225 | ↓* |
| 3 | 4-Hydroxybenzaldehyde | 121.029 | 3.6 | 0.3150 | 121;120;120 | [M-H]- | C7H6O2 | 1.1957 | 0.0176 | ↓* |
| 4 | Cholenate | 373.2733 | 3.9 | 0.4433 | 373.3;81;291.1 | [M-H]- | C24H38O3 | 1.0434 | 0.0356 | ↑* |
| 5 | Pyrocatechol | 109.0291 | 3.7 | 0.3183 | 109;108 | [M-H]- | C6H6O2 | 2.1148 | 0.0125 | ↓* |
| 6 | 3,5-Dimethoxybenzoic acid | 181.0496 | 5.4 | 0.5333 | 181.1;81;181 | [M-H]- | C9H10O4 | 1.3225 | 0.0178 | ↑* |
| 7 | Carnitine | 162.1115 | 6.3 | 3.3833 | 162.1;103;60.1 | [M+H]+ | C7H15NO3 | 2.3929 | 0.0152 | ↓* |
| 8 | Pyridoxine | 168.066 | 3.4 | 0.6650 | 150.1;123;168.1 | [M-H]- | C8H11NO3 | 1.5204 | 0.0219 | ↓* |
| 9 | Valproic acid | 143.1072 | 3.7 | 0.4383 | 143.1;71 | [M-H]- | C8H16O2 | 1.3427 | 0.0421 | ↓* |
| 10 | O-Acetylserine | 146.0454 | 3.3 | 2.8817 | 74;116;62 | [M-H]- | C5H9NO4 | 1.1679 | 0.0210 | ↑* |
| 11 | Fuziline | 454.2773 | 5.8 | 1.1417 | 454.3;436.3;50.5 | [M+H]+ | C24H39NO7 | 1.6691 | 0.0009 | ↓** |
| 12 | Mesaconitine | 632.3032 | 5.4 | 0.4183 | 632.3;572.3;105 | [M+H]+ | C33H45NO11 | 1.5169 | 0.0171 | ↓* |
| 13 | 3-Methylglutaric acid | 145.0502 | 3.2 | 3.6867 | 101;101.1;57 | [M-H]- | C6H10O4 | 1.0350 | 0.0116 | ↑* |
| 14 | Biotin | 245.094 | 5.8 | 2.6433 | 227.1;127.1;118.1 | [M+H]+ | C10H16N2O3S | 1.0126 | 0.0344 | ↑* |
| 15 | Uridine | 243.0614 | 3.4 | 1.1283 | 110;200.1;243.1 | [M-H]- | C9H12N2O6 | 1.9831 | 0.0148 | ↓* |
| 16 | Uridine 5'-monophosphate (UMP) | 323.0278 | 2.4 | 4.2817 | 79;97;323 | [M-H]- | C9H13N2O9P | 1.1076 | 0.0325 | ↓* |
| 17 | Biliverdin | 581.2384 | 3.7 | 2.4200 | 285.1;521.2;239.1 | [M-H]- | C33H34N4O6 | 1.0859 | 0.0494 | ↑* |
| 18 | 5-Methoxytryptamine | 191.1167 | 6.3 | 1.5717 | 190;162.1;191.1 | [M+H]+ | C11H14N2O | 1.1712 | 0.0389 | ↑* |
| 19 | Benzoylhypaconine | 574.2978 | 5.6 | 1.0350 | 574.3;542.3;63.8 | [M+H]+ | C31H43NO9 | 1.7605 | 0.0008 | ↑** |
| 20 | Nicotinamide adenine dinucleotide (NAD) | 664.1136 | 4.3 | 4.3250 | 136.1;428;112 | [M]+ | C21H28N7O14P2 | 1.6510 | 0.0155 | ↓* |
| 21 | Beiwutine | 648.2974 | 6.2 | 0.4983 | 648.3;588.3;72 | [M+H]+ | C33H45NO12 | 1.9869 | 0.0014 | ↓** |
| 22 | N,N-Diethyl-2-aminoethanol | 118.1219 | 6.5 | 2.2633 | 118.1;60.1;59 | [M+H]+ | C6H15NO | 1.4338 | 0.0052 | ↑** |
| 23 | Daltogen | 150.1115 | 6.6 | 0.9900 | 150.1;150.1;132.1 | [M+H]+ | C6H15NO3 | 1.1223 | 0.0119 | ↓* |
| 24 | 4-Hydroxyphenylpyruvic acid | 179.034 | 5.5 | 0.6767 | 179;80;135 | [M-H]- | C9H8O4 | 1.0304 | 0.0132 | ↑* |
| 25 | Benzoylaconine | 604.3079 | 6.2 | 1.1733 | 604.3;554.3;105 | [M+H]+ | C32H45NO10 | 2.0968 | 0.0012 | ↑** |
| 26 | N-Acetylserotonin | 219.1112 | 7.4 | 0.3883 | 160.1;219.1;142.1 | [M+H]+ | C12H14N2O2 | 1.0577 | 0.0094 | ↑** |
| 27 | PC(18:0/14:0) | 734.5648 | 6.3 | 1.4683 | 184.1;734.6;86.1 | [M+H]+ | C40H80NO8P | 1.5346 | 0.0329 | ↑* |
| 28 | Benzoylmesaconine | 590.2918 | 7.2 | 1.2583 | 590.3;540.3;65.6 | [M+H]+ | C31H43NO10 | 1.7251 | 0.0007 | ↑** |
| 29 | H-Val-Ala-OH | 189.1221 | 6.7 | 2.2983 | 189.1;189.1;112.1 | [M+H]+ | C8H16N2O3 | 1.8267 | 0.0013 | ↑** |
| 30 | MES | 194.0487 | 2.8 | 1.0817 | 151.1;95;194 | [M-H]- | C6H13NO4S | 1.7868 | 0.0159 | ↑* |
| 31 | 5-Oxooctanoic acid | 157.0864 | 3.5 | 0.6667 | 157.1;127;157 | [M-H]- | C8H14O3 | 1.1506 | 0.0404 | ↓* |
| 32 | Metoprolol acid | 268.1528 | 5.7 | 3.5317 | 268.2;267.1;167.1 | [M+H]+ | C14H21NO4 | 1.1314 | 0.0061 | ↑** |
| 33 | 2-Amino-4-methoxyphenol | 140.0697 | 6.7 | 0.5350 | 140.1;139.1;58.1 | [M+H]+ | C7H9NO2 | 1.0399 | 0.0466 | ↑* |
| 34 | 3-Methylxanthine | 165.0413 | 2.9 | 1.8567 | 165;121;121.1 | [M-H]- | C6H6N4O2 | 1.1379 | 0.0221 | ↑* |
| 35 | Ceritinib | 558.2303 | 0.5 | 0.4700 | 558.2;480.2;539.5 | [M+H]+ | C28H36ClN5O3S | 1.3471 | 0.0028 | ↓** |
| 36 | N-Tigloylglycine | 156.0661 | 3.3 | 2.3783 | 156.1;84;114.1 | [M-H]- | C7H11NO3 | 1.2577 | 0.0052 | ↑** |
| 37 | Bilirubin | 585.2691 | 2.9 | 0.4500 | 299.1;300.1;585.3 | [M+H]+ | C33H36N4O6 | 1.5066 | 0.0197 | ↑* |
| 38 | Fludarabine (phosphate) | 346.035 | 2.3 | 4.5683 | 203;59;201 | [M-H2O-H]- | C10H13FN5O7P | 1.9491 | 0.0449 | ↑* |
| 39 | 4-Methylquinolin-2-ol | 160.0747 | 6.2 | 0.8700 | 160;160.1;159.1 | [M+H]+ | C10H9NO | 1.4789 | 0.0068 | ↑** |
| 40 | Homovanillic acid | 181.05 | 3.2 | 1.9900 | 181;137.1;138.1 | [M-H]- | C9H10O4 | 1.5414 | 0.0143 | ↑* |
| 41 | Ibutilide (fumarate) | 365.2319 | 14.2 | 0.7583 | 365.2;317.2;319.2 | [M-H2O-H]- | C20H36N2O3S | 1.3870 | 0.0063 | ↑** |
| 42 | 1-Methyl-6-phenyl-1H-imidazo[4,5-b]pyridin-2-amine | 223.0967 | 9.7 | 0.8533 | 223.1;223.1;205.1 | [M-H]- | C13H12N4 | 1.2486 | 0.0137 | ↑* |
| 43 | Ribothymidine | 257.081 | 12.2 | 0.3183 | 256.2;257.1 | [M-H]- | C10H14N2O6 | 1.5259 | 0.0005 | ↑** |
